# Evolutionary diversity and novelty of DNA repair genes in asexual Bdelloid rotifers

**DOI:** 10.1186/s12862-018-1288-9

**Published:** 2018-11-28

**Authors:** Bette J. Hecox-Lea, David B. Mark Welch

**Affiliations:** 1 Josephine Bay Paul Center for Comparative Molecular Biology and Evolution, Marine Biological Laboratory, Woods Hole, MA USA; 2 Department of Biology, Northeastern University, Boston, MA USA

**Keywords:** AlkD, APLF, Blm, Fpg, Ku 70/80, Ligase K, NHEJ, Polymerase lambda, UVDE, XRCC4

## Abstract

**Background:**

Bdelloid rotifers are the oldest, most diverse and successful animal taxon for which males, hermaphrodites, and traditional meiosis are unknown. Their degenerate tetraploid genome, with 2–4 copies of most loci, includes thousands of genes acquired from all domains of life by horizontal transfer. Many bdelloid species thrive in ephemerally aquatic habitats by surviving desiccation at any life stage with no loss of fecundity or lifespan. Their unique genomic diversity and the intense selective pressure of desiccation provide an exceptional opportunity to study the evolution of diversity and novelty in genes involved in DNA repair.

**Results:**

We used genomic data and RNA-Seq of the desiccation process in the bdelloid *Adineta vaga* to characterize DNA damage reversal, translesion synthesis, and the major DNA repair pathways: base, nucleotide, and alternate excision repair, mismatch repair (MMR), and double strand break repair by homologous recombination (HR) and classical non-homologous end joining (NHEJ). We identify multiple horizontally transferred DNA damage response genes otherwise unknown in animals (AlkD, Fpg, LigK UVDE), and the presence of genes often considered vertebrate specific, particularly in the NHEJ complex and X family polymerases. While 75–100% of genes involved in MMR and HR are present in 0–2 copies, genes involved in NHEJ, which are present in only a single copy in nearly all other animals, are retained in 3–8 copies. We present structural predictions and expression evidence of neo- or sub-functionalization of multiple copy genes involved in NHEJ and other repair processes.

**Conclusion:**

The horizontally-acquired genes and duplicated genes in BER and NHEJ suggest resilience to oxidative damage is conferred in part by increased DNA damage recognition and efficient end repair capabilities. The pattern of gene loss and retention in MMR and HR may facilitate recombination and gene conversion between divergent sequences, thus providing at least some of the benefits of sex. The unique retention and divergence of duplicates genes in NHEJ may be facilitated by the lack of efficient selection in the absence of meiotic recombination and independent assortment, and may contribute to the evolutionary success of bdelloids.

**Electronic supplementary material:**

The online version of this article (10.1186/s12862-018-1288-9) contains supplementary material, which is available to authorized users.

## Background

Bdelloid rotifers are a diverse class of over 450 species of desiccation-tolerant, radiation-resistant, asexual microinvertebrates that inhabit diverse aquatic and limnoterrestrial habitats around the globe. Populations are wholly composed of parthenogenic females, with no evidence of males or hermaphrodites. Bdelloids reproduce through mitotic division of oocyte mother cells, with no chromosome pairing or reduction, producing clonal offspring [1, 2]. While evidence of some form of genetic exchange has been reported [3] the mechanism remains unknown and the group is generally considered to have evolved without sexual reproduction and to be the largest, most successful obligately asexual animal taxon [4–6].

The genome structure of bdelloid rotifers is degenerate tetraploid [5, 7, 8]. Under this model, a whole genome duplication in a common bdelloid ancestor created a tetraploid with two copies (ohnologs) of the genome. Within each ohnologous diploid set, (former) alleles diverged due to the lack of meiotic independent segregation and syngamy [9], but may also be routinely homogenized by gene conversion [5, 8]. Ohnologs and former alleles have diverged by an average of 27% and 1% at non-synonymous positions, respectively. Gene copies have been lost over time, so that roughly 40% of genes are still present in four copies. Additionally, 5–10% of bdelloid genes have been horizontally-acquired from non-metazoans, and the horizontal gene transfer (HGT) process is both ancient and ongoing. The horizontally-acquired, or “alien” genes are transcriptionally active and properly spliced, and produce functional proteins [5, 10–12].

Bdelloids can enter a state of anhydrobiosis at any life stage in response to desiccation, an ability first recognized by Leeuwenhoek more than 300 years ago [13]. When water in the environment evaporates, a bdelloid loses nearly all unbound water, reducing its weight by 95% and effectively ceasing metabolism; when water returns to the environment, the bdelloid hydrates and resumes activity with no loss of lifespan or fecundity [14–17]. Bdelloid species that inhabit desiccation-prone environments enter anhydrobiosis on average once per generation [18, 19]. That bdelloids have adapted to this condition can be seen in the higher fecundity and increased total fitness of bdelloid populations that have been through repeated rounds of anhydrobiosis compared to those that remain constantly hydrated [20, 21].

The tolerance of bdelloids to desiccation likely underpins their extreme resistance to ionizing radiation (IR). Even when hydrated, exposure to 600 Gy—well in excess of the lethal dose for most other animals—causes minimal reduction of fertility in exposed mothers or their offspring [22]. In bdelloids, resistance to the damage of anhydrobiosis and IR appears to rely on antioxidants rather than osmoprotectants such as trehalose [23–25], and damage resilience is seen in the DNA double strand break (DSB) repair during recovery from desiccation and IR [22, 26–30].

The extreme oxidizing conditions of desiccation and IR produce closely spaced DNA lesions that can lead to strand breaks. DSBs are a late stage of oxidative damage, with an estimated 25 single strand breaks for every observed DSB [31, 36]; thus, substantial additional DNA damage underlies the DSBs found in previous studies [22, 29]. Further, oxidatively-caused lesions create DNA ends with a variety of chemical alterations that can block DNA synthesis and/or ligation, the final two repair steps [31–33]. Such chemical blockages are removed by the end-processing enzymes of base excision repair and non-homologous end-joining, the main pathways for mending oxidatively-damaged bases and oxidatively-broken DNA, respectively.

Given the many unique aspects of bdelloid biology, we reasoned that understanding the evolution and function of bdelloid DNA damage response (DDR) genes would reveal functions selected for in oxidative damage resilience, and suggest mechanisms that may contribute genetic novelty in the absence of sex, helping to explain the persistent mystery of the success of bdelloid rotifers. Therefore, we identified and analyzed the multiple copies of DDR genes in the bdelloid *Adineta vaga*, defined as components of direct damage reversal, DNA repair, and damage tolerance by translesion synthesis (TLS) polymerases.

Direct damage reversal describes the removal of certain covalent adducts to DNA, particularly pyrimidine dimers and small alkyl groups bound to guanine, by single proteins without excising a base or incising the DNA backbone [34]. DNA repair occurs through excision repair, with DSBs repaired by homologous recombination or non-homologous end joining. When unrepaired damage is encountered during DNA replication, the replicative polymerase may be replaced by a lower fidelity, error-prone TLS polymerase to continue synthesis across the damaged template, leaving it unrepaired [35].

There are four excision repair pathways that correct damaged or mismatched bases by lesion excision and DNA backbone incision, repair synthesis, and ligation [36]. Base excision repair (BER) removes small, subtle base lesions caused by oxidation, alkylation, deamination or base loss. Nucleotide excision repair (NER) recognizes and removes bulky, helix-distorting lesions often caused by UV or alkylation. Mismatch repair (MMR) removes misincorporated bases and indel-caused small loops. Alternative excision repair (AER) is initiated by a single endonuclease incision and completed with downstream mechanisms of NER, BER or single-strand break repair [37].

Homologous recombination (HR) is a high-fidelity, template-dependent repair system that processes DSBs and DNA gaps and can lead to crossing-over and/or gene conversion [38, 39]. MMR is spatially and temporally coupled with HR, and largely prevents divergent sequences from participating in HR. Classical non-homologous end-joining (NHEJ) is an extremely robust, mechanistically flexible repair path that binds and stabilizes broken ends, repairs ends, fills gaps and ligates [31]. NHEJ is primarily responsible for repairing breaks induced by oxidation because it has extensive capabilities to repair complex chemical damages at the broken ends [31, 40].

The synthesis step of most repair pathways is carried out by high-fidelity, high-processivity replicative polymerases. Synthesis as characterized in vertebrate short-patch BER and NHEJ is primarily performed by the X-family polymerases DNA polymerase beta (Polβ) and lambda (Polλ), respectively. Polβ and Polλ are lower-processivity and lower-fidelity gap-filling polymerases that have lyase domains to remove ligation-blocking groups often left at 5’ends in these pathways.

The results of our DDR inventory and of differential gene expression entering and recovering from desiccation focused our analyses primarily on DNA repair pathways rather than reversal or tolerance. We present evidence of the retention of genes often considered vertebrate-specific, horizontal gene transfer of genes novel to metazoans, the loss of some MMR and HR genes, expansion and diversification of NHEJ genes, and functional divergence based on codon differences, predicted protein structure, and differential gene expression during desiccation.

## Results

**Fig. 1:**
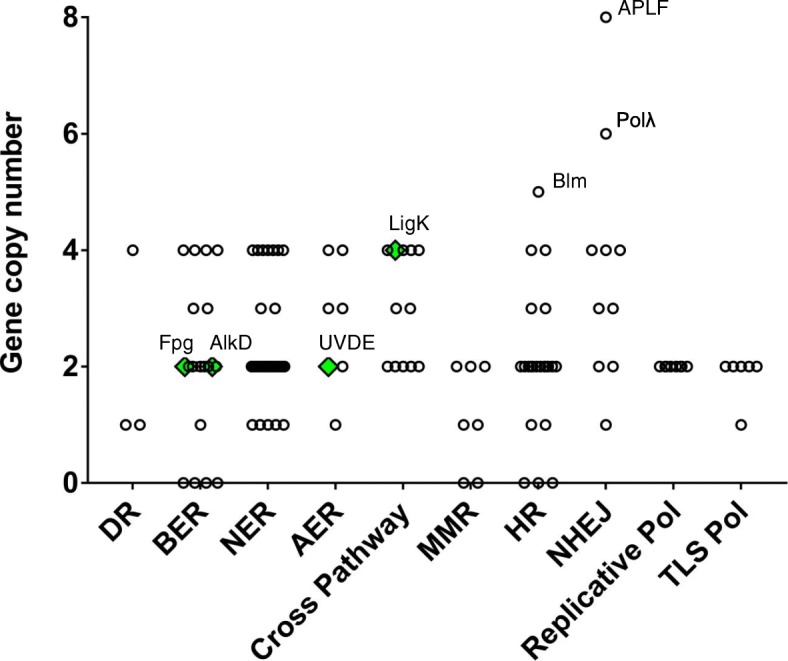
Gene Copy Number per Gene by DDR Category. Metazoan genes are indicated with open circles; non-metazoan genes by solid green diamonds. DR: Direct Reversal; BER: Base Excision Repair; NER: Nucleotide Excision Repair; AER: Alternate Excision Repair; MMR: Mismatch Repair; HR: Homologous Recombination; NHEJ: Non-homologous End Joining; TLS: Translesion Synthesis; Pol: Polymerase

We examined the *Adineta vaga* genome for 116 major conserved metazoan genes involved in direct damage reversal repair, BER, NER, AER, MMR, HR, NHEJ, and replicative and translesion synthesis. We identified 107 of these genes representing a total of 270 gene copies (Fig. 1, Table 1, Additional file 1). We also identified four genes, three present in two copies and one in four copies, that have been acquired from non-metazoan sources. Through manual editing and RNA-Seq data mapping we improved the annotation of 58 copies. As shown in Fig. 1, gene copy number varied from zero to eight, with most genes present as either a pair of former alleles (2 copies) or a pair of allele pairs (4 copies) representing ohnologs of the ancestral genome duplication [5, 8]. Genes for which we identified an odd number of copies may have two identical copies that collapsed into a single contig during genome assembly, or may have a hemizygous copy due to deletion or decay of the allelic copy. Retention of ohnologous copies is highly variable across DDR pathways, ranging from zero in MMR and among replicative and trans-lesion polymerases, to 70% in NHEJ.

**Table 1:** Major Conserved Metazoan DDR Genes

Gene	Gene Description	KO	#	Cel	Dme	Hsa
Direct Reversal (DR)						
PHRB	deoxyribodipyrimidine photo lyase	K01669	4		X	
ALKBH1	alkylated DNA repair protein	K10765	1	X	X	X
MGMT	alkylated DNA repair protein	K00567	1	X	X	X
Base Excision Repair (BER)						
FPG	formamidopyrimidine-DNA glycosylase	K10563	2			
NEIL1/2/3	endonuclease VIII like 1,2, or 3	K10567/8/9	0			X
MPG	DNA-3-methyladenine glycosylase	K03652	0			X
OGG1	8-oxoguanine DNA glycosylase	K03660	3		X	X
AlkD	DNA alkylation repair enzyme	K00000	2			
MUTYH	mutY homolog	K03575	2			X
SMUG1	ss-selective monofunctional uracil DNA glycosylase	K10800	0			X
MBD4	methyl-CpG-binding domain protein 4	K10801	0			X
NTHL1	nth endonuclease III-like 1	K10773	1	X	X	X
TDG/MUG	TDG/ MUG DNA glycosylase family protein	K20813	4		X	X
UNG1	mitochondrial uracil DNA glycosylase	K03648	2	X		X
UNG2	nuclear uracil DNA glycosylase	K03648	4	X		X
APEX1	AP endonuclease 1	K10771	4	X	X	X
APEX2	AP endonuclease 2	K10772	2			X
PARP1	poly (ADP-ribose) polymerase family, member 1	K10798	4	X	X	X
XRCC1	X-ray repair complementing defective repair in Chinese hamster cells 1	K10803	2		X	X
TDP1	tyrosyl-DNA phosphodiesterase 1	K10862	2		X	X
FEN1	flap endonuclease-1	K04799	3	X	X	X
POLB	DNA polymerase beta	K02330	2			X
LIG3	DNA ligase 3	K10776	2			X
Nucleotide Excision Repair (NER)						
RBX1	RING-box protein 1	K03868	4	X	X	X
CUL4	cullin 4	K10609	4	X	X	X
DDB1, XPE	xeroderma pigmentosum group E-complementing protein	K10610	3	X	X	X
DDB2	DNA damage binding protein 2	K10140	2			X
CSA, ERCC8	excision repair cross-complementing rodent repair deficiency, complementation group 8	K10570	2	X	X	X
XPC	xeroderma pigmentosum group C-complementing protein	K10838	2	X	X	X
RAD23	UV excision repair protein RAD23	K10839	4	X	X	X
CETN2	centrin-2	K10840	4			X
XPA	xeroderma pigmentosum group A-complementing protein	K10847	2	X	X	X
ERCC1	excision repair cross-complementing rodent repair deficiency, complementation group 1	K10849	2	X	X	X
ERCC2, XPD	xeroderma pigmentosum group D-complementing protein	K10844	2	X	X	X
ERCC3, XPB	xeroderma pigmentosum group B-complementing protein	K10843	3	X	X	X
ERCC4, XPF	xeroderma pigmentosum group F-complementing protein	K10848	2	X	X	X
ERCC5, XPG	xeroderma pigmentosum group G-complementing protein	K10846	2	X	X	X
ERCC6, CSB	excision repair cross-complementing rodent repair deficiency, complementation group 6	K10841	2	X		X
TFIIH1	transcription initiation factor TFIIH subunit 1	K03141	1	X	X	X
TFIIH2	transcription initiation factor TFIIH subunit 2	K03142	2	X	X	X
TFIIH3	transcription initiation factor TFIIH subunit 3	K03143	1	X	X	X
TFIIH4	transcription initiation factor TFIIH subunit 4	K03144	1	X	X	X
MMS19	DNA repair/transcription protein MET18/MMS19	K15075	1	X	X	X
RPB1	DNA-directed RNA polymerase II subunit RPB1	K03006	4	X	X	X
RPB2	DNA-directed RNA polymerase II subunit RPB2	K03010	2	X	X	X
RPB3	DNA-directed RNA polymerase II subunit RPB3	K03011	1	X	X	X
RPB4	DNA-directed RNA polymerase II subunit RPB4	K03012	2	X	X	X
RPB5	DNA-directed RNA polymerases I, II, and III subunit RPABC1	K03013	4	X	X	X
RPB6	DNA-directed RNA polymerases I, II, and III subunit RPABC2	K03014	4	X	X	X
RPB7	DNA-directed RNA polymerase II subunit RPB7	K03015	2	X	X	X
RPB8	DNA-directed RNA polymerases I, II, and III subunit RPABC3	K03016	2	X	X	X
RPB11	DNA-directed RNA polymerase II subunit RPB11	K03008	2	X	X	X
RPB12	DNA-directed RNA polymerases I, II, and III subunit RPABC4	K03009	2	X	X	X
Alternative Excision Repair (AER)						
UVDE	UV DNA damage endonuclease	K13281	2			
SMC6	structural maintenance of chromosomes 6	K22804	4		X	X
RAD51	DNA repair protein RAD51	K04482	4	X	X	X
RAD54	DNA repair and recombination protein RAD54 and RAD54-like protein	K10875	2	X	X	X
RAD54L2	RAD54-like protein 2	K10876	3	X	X	X
EXO1	exonuclease 1	K10746	2	X	X	X
FEN1	flap endonuclease-1	K04799	3	X	X	X
Cross Pathway						
LIG1	DNA ligase 1	K10747	2	X	X	X
LIGK	DNA ligase (ATP)	K00000	4			
PCNA	proliferating cell nuclear antigen	K04802	4	X	X	X
PNKP	bifunctional polynucleotide phosphatase/kinase	K08073	4	X	X	X
HMGB1	high mobility group protein B1	K10802	2		X	X
RFC1	replication factor C subunit 1	K10754	4	X	X	X
RFC2	replication factor C subunit 2 of RFC2_4	K10755	4	X	X	X
RFC4	replication factor C subunit 4 of RFC2_4	K10755	2	X	X	X
RFC3_5	replication factor C subunit 3_5	K10756	2	X	X	X
RPA1	replication protein A1	K07466	4	X	X	X
RPA2	replication protein A2	K10739	3	X	X	X
RPA3	replication protein A3	K10740	2		X	X
Mismatch Repair (MMR)						
MSH2	DNA mismatch repair protein MSH2	K08735	1	X	X	X
MSH3	DNA mismatch repair protein MSH3	K08736	0			X
MSH6	DNA mismatch repair protein MSH6	K08737	2	X	X	X
MSH4	DNA mismatch repair protein MSH4, canonically meiotic	K08740	2	X	X	X
MSH5	DNA mismatch repair protein MSH5, canonically meiotic	K08741	2	X	X	X
PMS2	DNA mismatch repair protein PMS2	K10858	2	X	X	X
MLH1	DNA mismatch repair protein MLH1	K08734	2	X	X	X
MLH3	DNA mismatch repair protein MLH3	K08739	0			X
EXO1	exonuclease 1	K10746	2	X	X	X
Homologous Recombination (HR)						
RAD50	DNA repair protein RAD50	K10866	4	X	X	X
MRE11	double-strand break repair protein MRE11	K10865	2	X	X	X
NBS1	nibrin	K10867	0		X	X
ATM	ataxia telangectasia mutated family protein	K04728	2	X	X	X
RAD51	DNA repair protein RAD51	K04482	4	X	X	X
RAD51L1	RAD51-like protein 1	K10869	2			X
RAD51L2	RAD51-like protein 2	K10870	2		X	X
RAD52	DNA repair protein RAD52	K10873	0			X
BRCA2	breast cancer 2 susceptibility protein	K08775	0			X
RAD54	DNA repair and recombination protein RAD54 and RAD54-like protein	K10875	2	X	X	X
RAD54L2	RAD54-like protein 2	K10876	3	X	X	X
EME1	crossover junction endonuclease EME1	K10882	1		X	X
MUS81	crossover junction endonuclease MUS81	K08991	2	X	X	X
RECQ1	ATP-dependent DNA helicase Q1	K10899	4	X		X
BLM	Bloom’s syndrome DNA helicase	K10901	5	X	X	X
RECQL5	ATP-dependent DNA helicase Q5	K10902	2	X	X	X
RMI1	RecQ-mediated genome instability protein 1	K10990	1	X		X
TOP3A	DNA topoisomerase 3 alpha	K03165	2	X	X	X
TOP3B	DNA topoisomerase 3 beta	K03165	2	X	X	X
SLX1	structure specific endonuclease subunit SLX1	K15078	2	X	X	X
SLX4	structure-specific endonuclease subunit SLX4	K10484	2			X
GEN1	Gen homolog 1, endonuclease	K15338	2	X	X	X
Non-Homologous End Joining (NHEJ)						
KU70	ATP-dependent DNA helicase 2 subunit 1	K10884	4	X	X	X
KU80	ATP-dependent DNA helicase 2 subunit 2	K10885	4	X	X	X
DNAPKcs	DNA-dependent protein kinase catalytic subunit	K06642	3			X
ARTEMIS	DNA cross-link repair 1C protein	K10887	4			X
APTX	aprataxin	K10863	2		X	X
APLF	aprataxin and PNK-like factor	K13295	8		X	X
POLL	DNA polymerase lambda	K03512	6			X
XLF	non-homologous end-joining factor 1	K10980	2			X
XRCC4	DNA-repair protein XRCC4	K10886	3			X
LIG4	DNA ligase 4	K10777	1			X
Replicative Polymerases (Pol)						
POLA1	DNA polymerase alpha subunit A	K02320	2	X	X	X
POLA2	DNA polymerase alpha subunit B	K02321	2	X	X	X
POLD1	DNA polymerase delta subunit 1	K02327	2	X	X	X
POLD2	DNA polymerase delta subunit 2	K02328	2	X	X	X
POLE1	DNA polymerase epsilon subunit 1	K02324	2	X	X	X
POLE2	DNA polymerase epsilon subunit 2	K02325	2	X	X	X
POLG1	DNA polymerase gamma 1	K02332	2	X	X	X
POLG2	DNA polymerase gamma 2	K02333	2		X	X
Translesion Synthesis (TLS) Polymerases (Pol)						
POLH	DNA polymerase eta	K03509	2	X	X	X
POLK	DNA polymerase kappa	K03511	2	X		X
POLQ	DNA polymerase theta	K02349	1	X	X	X
REV1	DNA polymerase zeta, Rev1 subunit	K03515	2	X	X	X
REV7	DNA polymerase zeta, Rev7 subunit	K03508	2			X
REV3L	DNA polymerase zeta, Rev3-like subunit	K02350	2	X	X	X

Genes are classified into the ten categories shown in Fig. 1; some genes appear in more than one category. See Additional file 1 for specific *A. vaga* locus identifiers and other details. KO: KEGG Ontology accession; #: copy number in *A. vaga*; Cel: *C. elegans*; Dme: *D. melanogaster*; Hsa: *H. sapiens*. An “X” indicates an ortholog is present in the KEGG Orthology Database for the indicated species

We identified 48 of the 52 major conserved genes associated in eukaryotes with excision repair. The four missing genes, MBD4, MPG, NEI-like, and SMUG1, are BER glycosylases with redundant or overlapping function with others, and it is not unusual for any one of them to be absent from a metazoan. Glycosylases are a diverse, relatively specialized suite of enzymes that typically perform a methodical search to recognize subtle alterations in the DNA duplex caused by damaged bases, and excise those bases, initiating BER. The MMR pathway is conspicuous for the absence of ohnologs and two orthologs, Mlh3 and Msh3. One or both of these genes are absent in many animals (*Caenorhabditis elegans* and *Drosophila* lack both). Monogonont rotifers also appear to lack Msh3, but do have Mlh3 [41], indicating a relatively recent loss in bdelloids. More than three quarters of the genes involved in HR lack ohnologs, and three conserved genes are missing: Nbs1, Rad52, and BRCA2. Nbs1 and Rad 52 are present in monogonont rotifers [41], but absent in many invertebrates (*C. elegans* lacks Nbs1 and both *Drosophila* and *C. elegans* lack Rad52). Most animals, including monogonont rotifers and other Lophotrochozoans, have maintained BRCA2.

More than half of the genes we investigated are significantly down-regulated during entry into desiccation or recovery from desiccation, or both. Only 8% of the genes are significantly up-regulated in either or both entry and recovery from desiccation. All gene copies, designations, genome coordinates, expression levels, and results of differential expression significance tests are listed in Additional file 1.

### Horizontal transfer of excision repair genes

All four horizontally transferred genes we identified are involved in excision repair: formamidopyrimidine DNA glycosylase (Fpg), alkylpurine DNA glycosylase (AlkD), ultraviolet damage endonuclease (UVDE), and a kinetoplastid ATP-dependent polynucleotide ligase (LigK), classified in the cross pathway set in Fig. 1. There is evidence of all four genes in transcriptome data from other bdelloid species and no evidence that these genes are present in genome or transcriptome data from two monogonont species (not shown), thus these genes appear to have been acquired early in bdelloid evolution.

#### Fpg

**Fig. 2:**
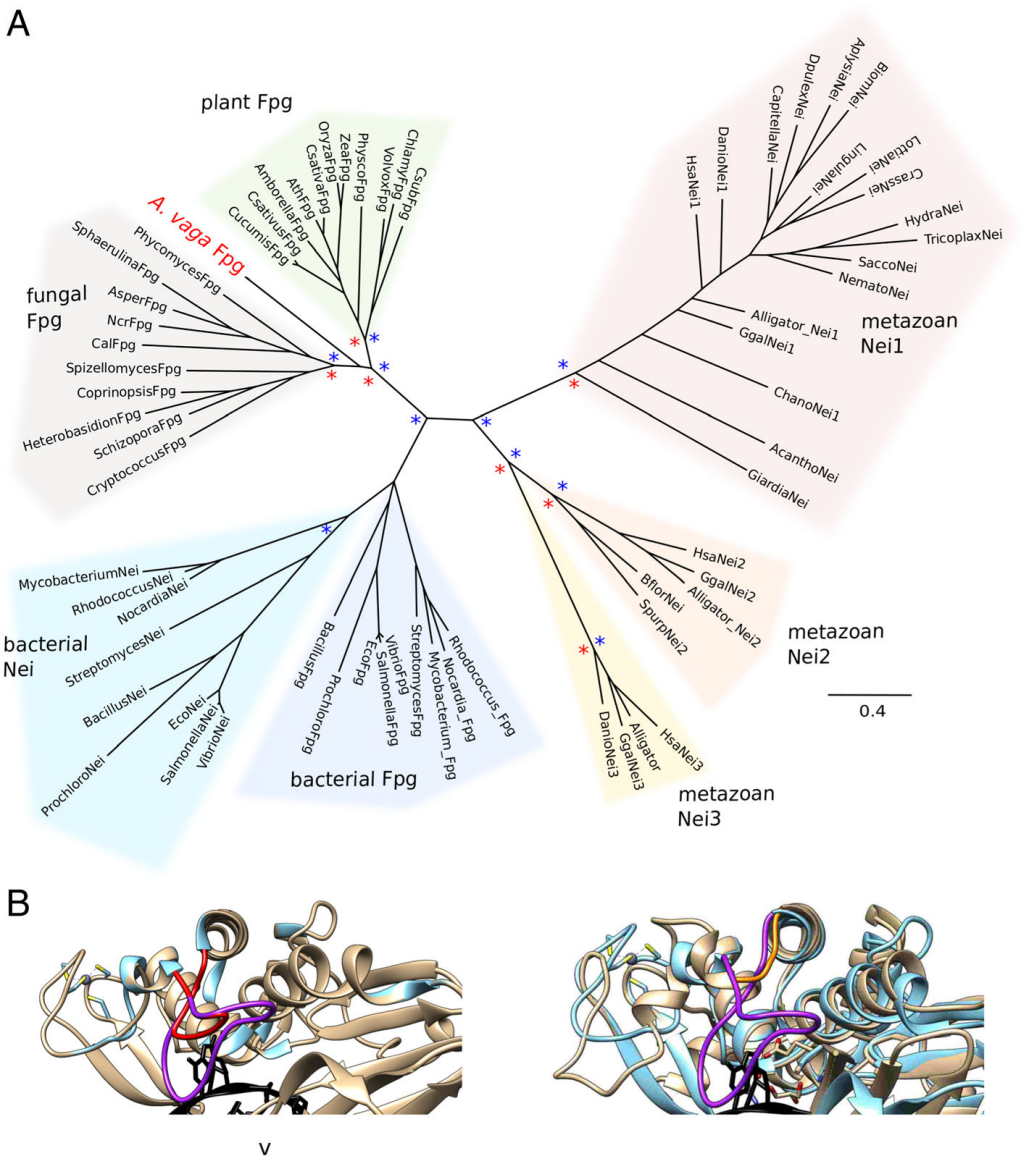
Fpg. **a** Phylogeny of Fpg and Nei genes; *Adineta vaga* Fpg is in red. Clades with greater than 70% RAxML bootstrap support or 90% MrBayes posterior probability are marked with red and blue asterisks, respectively. Complete trees and accession numbers and species names of OTUs are available in Additional file 2. **b** The 8-oxoG capping loop region of Fpg (DNA shown in black). *Left*, *A. vaga* (AvFpg) in bronze threaded onto *Geobacillus stearothermophilus* Fpg (BstFpg, from *Bacillus* basonym, PDB 1R2Y) in blue. The BstFpg αF-β9/10 loop (purple) extends down to cover and trap the 8-oxoG, but AvFpg αF-β9/10 loop (red), is predicted to be too short to fully cover an 8-oxoG in the binding pocket. *Right*, *Arabidopsis thaliana* Fpg (AthFpg, PDB: 3TWK) in bronze overlaying BstFpg in blue, as shown in [43]. Here, the much shorter AthFpg αF-β9/10 loop (orange) cannot trap 8-oxoG in the binding pocket as the BstFpg loop (purple) can

*Adineta vaga* has a pair of Fpg genes likely acquired from a fungus (Fig. 2a). This is the only known occurrence of Fpg in an animal. Fpg and endonuclease VIII (Nei) compose a family of glycosylases that perform the first two steps in BER: base excision and incision of the DNA backbone. Eubacteria possess both Fpg and Nei, which have different substrate specificities. Within eukaryotes, Nei (Nei-like, NEIL) is known only in some metazoans (primarily deuterostomes) and some protists, while Fpg is known only in some plants and fungi [42].

Bacterial Fpg is an extensively-studied functional analog of eukaryotic Ogg1, primarily recognizing the most common nucleotide oxidation product, 7,8-dihydro-8-oxoguanine (8-oxoG). In contrast, eukaryotic Fpg does not recognize 8-oxoG, rather it recognizes abasic sites, formamidopyrimidines, and two late-stage oxidation products of 8-oxoG: guanidinohydantoin and spiroiminodihydantoin [42, 43]. The substrate difference between bacterial and eukaryotic Fpg homologs has been attributed to the eukaryotic Fpg’s shorter αF-β9/10 loop, also called the 8-oxoG capping loop, which cannot cap and retain an 8-oxoG in the active site [43]. Our structural comparisons of Fpg proteins from *A. vaga*, with those *Geobacillus stearothermophilus,* and *Arabidopsis thaliana,* as examined by [43]*,* predict the *A. vaga* Fpg αF-β9/10 loop is intermediate sized (Fig. 2b). This suggests that *A. vaga* Fpg cannot cap 8-oxoG completely and its substrate affinities may be different from those of previously characterized homologs. Fpg is one of the more abundant glycosylase transcripts in the *A. vaga* transcriptome across all conditions tested.

#### UVDE

**Fig. 3:**
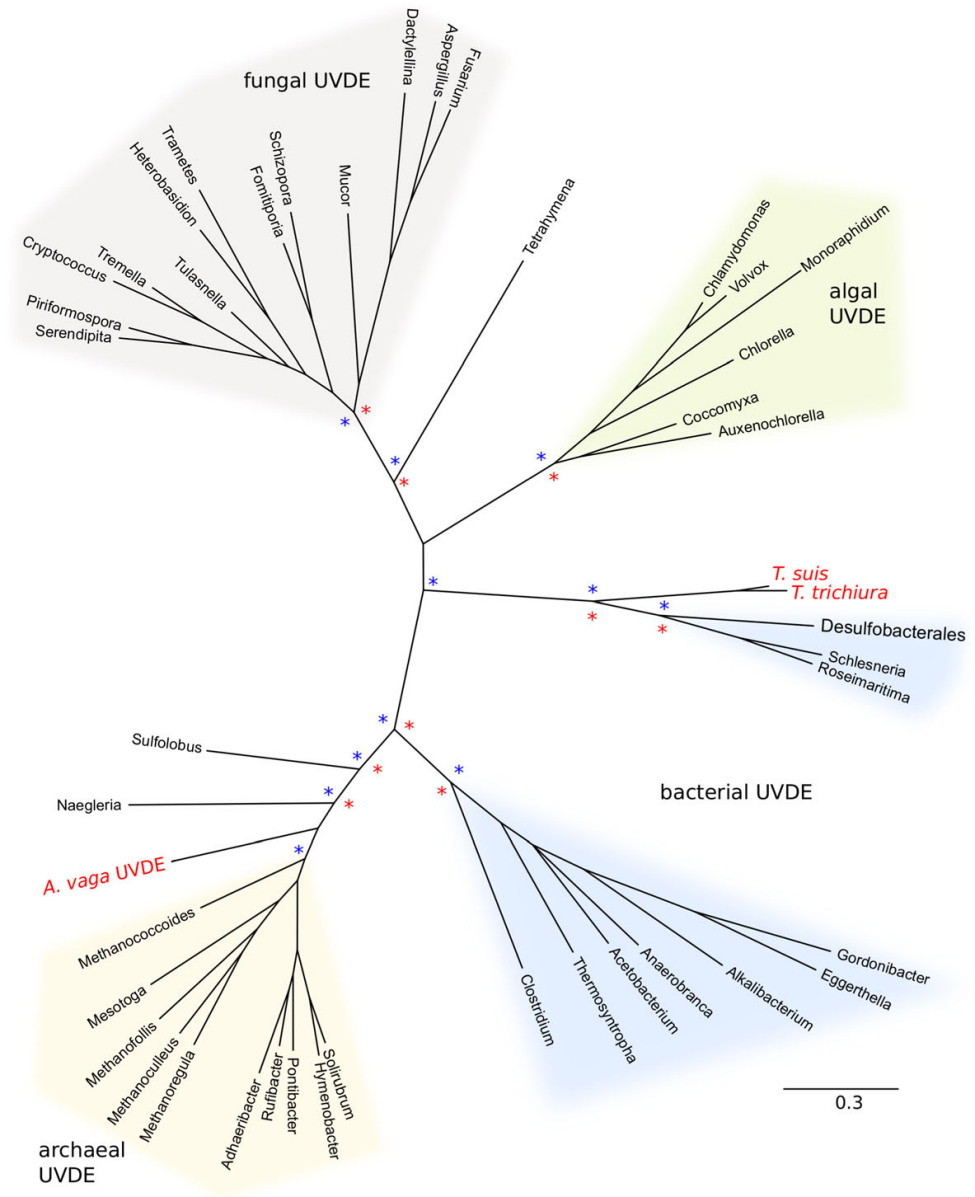
Phylogeny of UVDE. *Adineta vaga* and *Trichuris* ssp. Fpg are in red. Clades with greater than 70% RAxML bootstrap support or 90% MrBayes posterior probability are marked with red and blue asterisks, respectively. Complete trees and species names and accession numbers of OTUs are available in Additional file 2

*Adineta vaga* has a pair of UVDE genes likely acquired from an archaeon (Fig. 3). The only non-bdelloid animals with a UVDE-like gene in GenBank are two species of *Trichuris,* a genus of parasitic nematodes. Our phylogenetic analysis suggests that bdelloids and *Trichuris* represent independent occurrences of horizontal transfer of UVDE from different non-eukaryotic origins.

UVDE is an ATP-independent endonuclease that initiates AER. It recognizes and removes cyclobutane pyrimidine dimers (CPD) and 6–4 photoproducts (6–4)PP, both UV-induced damages normally recognized in eukaryotes by an approximately 30- protein recognition complex of NER. UVDE incises the DNA backbone 5′ to the damage, leaving a 5′ phosphate and 3′ hydroxyl ready for synthesis and ligation [37, 44, 45]. Evidence from in vitro and in vivo studies suggest UVDE can recognize a range of substrates typically targeted by NER, BER, or MMR including abasic sites, platinum adducts, uracil, dihydrouracil, abasic sites, mismatches, 3′-blocking groups, and short loops [46–54]. *A. vaga* UVDE is expressed at a low level under both hydrated and desiccating conditions.

#### AlkD

**Fig. 4:**
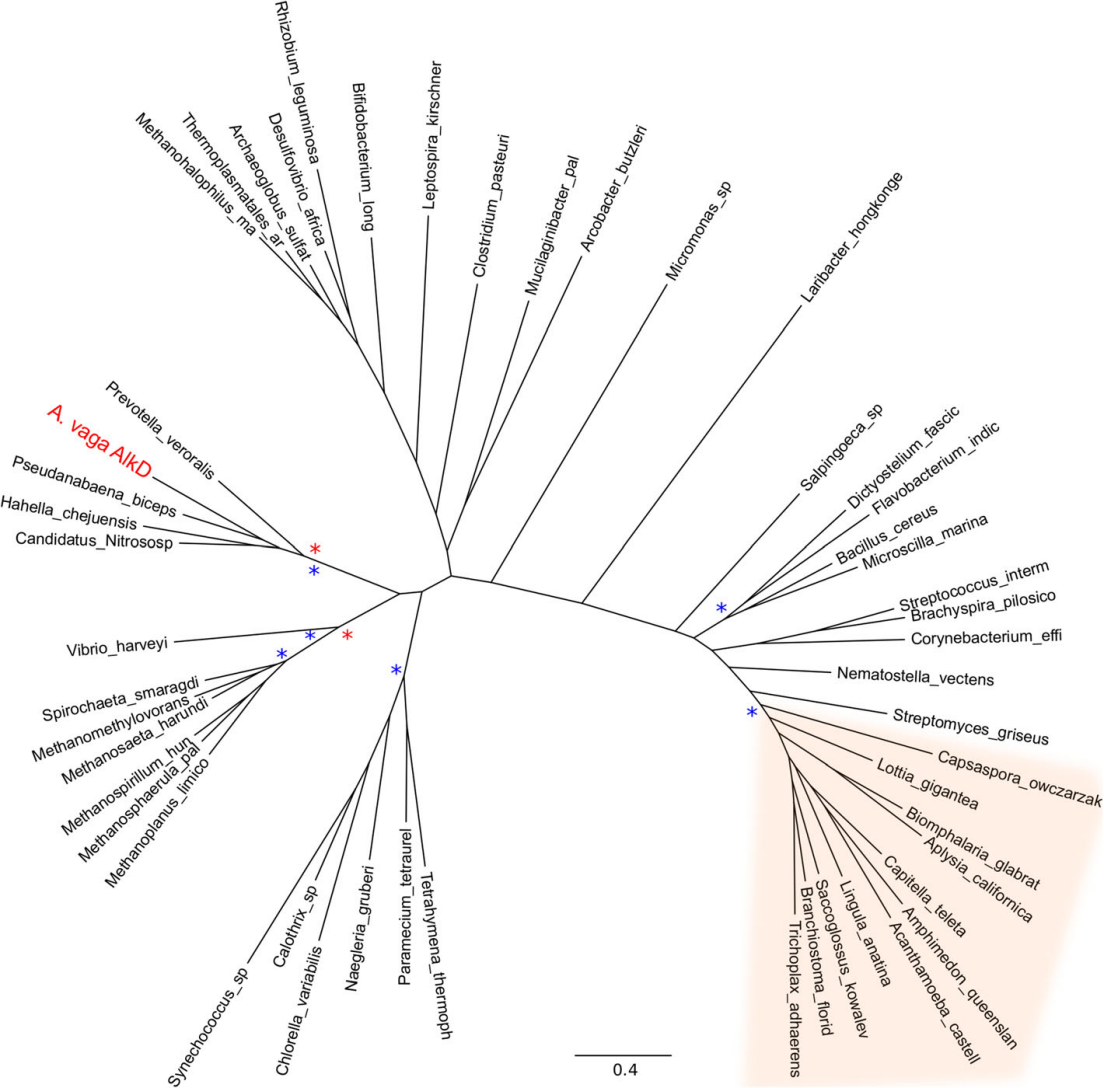
Phylogeny of AlkD. *Adineta vaga* AlkD is in red, well-separated from the clade of metazoan sequences, shaded in orange. Clades with greater than 70% RAxML bootstrap support or 90% MrBayes posterior probability are marked with red and blue asterisks, respectively. Complete trees and species names accession numbers of OTUs are available in Additional file 2

*Adineta vaga* has a pair of AlkD genes likely acquired from bacteria (Fig. 4). AlkD is an unusual glycosylase that recognizes damage without intercalation or base-flipping and excises both inherently unstable cationic alkylation damage, particularly N3- and N7-alkylpurines, and large alkylation adducts normally repaired by NER [55, 56]. An AlkD ortholog is sporadically present in Metazoa and has not been identified in vertebrates or ecdysozoans. We have not found a metazoan AlkD ortholog in any rotifer, and the *A. vaga* AlkD is clearly distinct from the metazoan AlkD lineage (Fig. 4). AlkD is the only BER glycosylase that is upregulated in *A. vaga* entering desiccation.

#### Ligase K

**Fig. 5:**
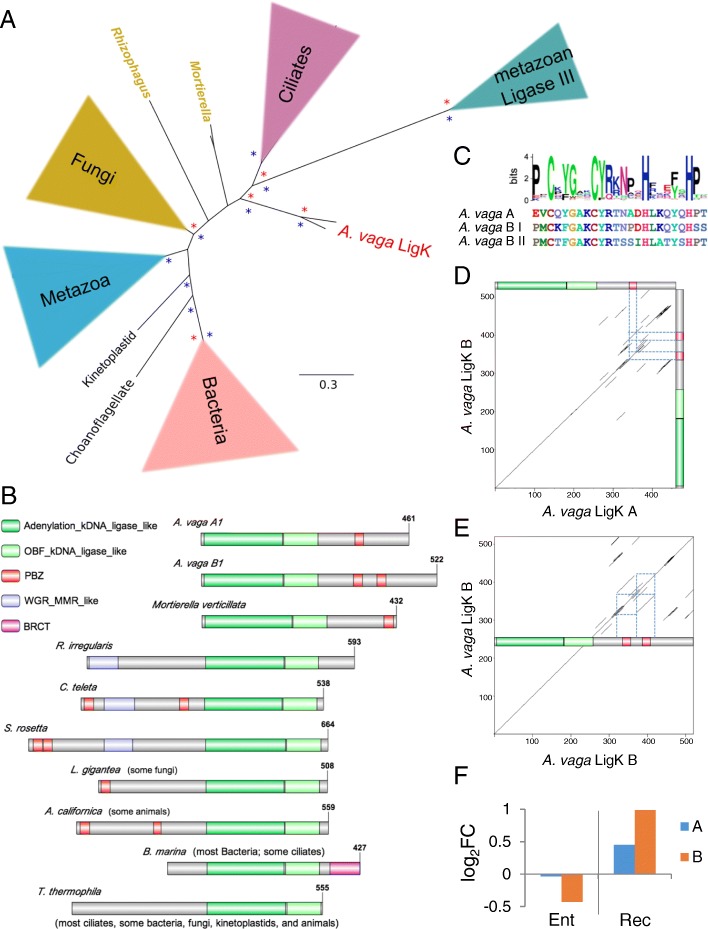
Ligase K. **a** Simplified phylogenetic tree of Ligase K with Ligase III as an outgroup; *Adineta vaga* Ligase K is in red. Clades with greater than 70% RAxML bootstrap support or 90% MrBayes posterior probability are marked with red and blue asterisks, respectively. Complete trees and species names and accession numbers of OTUs are available in Additional file 2. **b** Domain models of *A. vaga* Ligase K copies A1 and B1, with domain models of Ligase K peptides from other species (*Mortierella verticillata* KFH62561.1, *Rhizophagus irregularis* ESA15105.1, *Capitella teleta* ELT89513.1, *Salpingoeca rosetta* XP_004993722.1, *Lottia gigantean* XP_009061413.1, *Aplysia californica* XP_005100834.1, *Blastopirellula marina* WP_002650560.1, *Tetrahymena thermophila* XP_001011861.1). **c**) Comparison of *A. vaga* Ligase K PBZ domains with sequence logo of the Pfam model. **d, e** Dotplots generated with EMBOSS dotmatcher of copies B1 vs A1 and B1 to itself; lines along the diagonal indicate regions of similarity between the compares sequences. **f** Differential expression of *A. vaga* Ligase K ohnologs entering and recovering from desiccation, compared to hydrated controls. Values are log_2_ fold change of normalized counts, significance test values are listed in Additional file 1

*Adineta vaga* has a two ohnologous pairs of a unique form of DNA Ligase K (LigK, Fig. 5). LigK is an ATP-dependent polynucleotide ligase with a specific class of adenylation and OBF domains, both of which contain multiple DNA binding sites. Ligase K was named for its original characterization in kinetoplastids [57], and composes a distinct evolutionary lineage from eukaryotic Lig1 and Lig3/4. We have identified LigK throughout Eubacteria, in many fungi (though not *Saccharomyces*, *Schizosaccharomycetes*, or *Neurospora*)*,* and sporadically in protists and Metazoa (though not in vertebrates, arthropods, or nematodes). The *A. vaga* LigK ohnologs are phylogenetically distinct from those in other Metazoa (Fig. 5a).

LigK contains a core of kDNA-adenylation and kDNA-OBF domains, usually at the C terminus, and generally contain other domains, such as poly(ADP-ribose) (PAR)-binding zinc finger (PBZ) domains, also called APLF-like zinc fingers, located on the N terminal side of the core domains. PBZ domains are strongly associated with nuclease activity and DNA metabolism, and possibly histone interactions [58, 59]. Each *A. vaga* ohnolog has a domain organization not seen in any other metazoan: the core domains occur at the N terminus, followed by a long disordered region containing one (ohnolog A) or two (ohnolog B) PBZ domains surrounded by a series of proline-serine-threonine rich degenerate repeats (Fig. 5b,c). The only other characterized LigK gene with a PBZ domain on the carboxy side of the core is from the soil fungus *Mortierella*, which lacks the serine-threonine rich degenerate repeats and has the PBZ domain at the C terminus. The difference in the number of PBZ domains between the *A. vaga* A and B ohnologs is likely due to expansion/contraction facilitated by PST-rich repeats rather than degeneration of a domain (Fig. 5d,e). The A and B ohnologs of LigK differ in their expression pattern during desiccation: transcript levels of ohnolog A do not change with desiccation, while transcript levels of ohnolog B increase significantly during recovery from desiccation (Fig. 5f).

### Divergence of multiple copy DSB repair genes

Conspicuous among DDR genes in *A. vaga* are three present as duplicated ohnologs (more than 4 copies): Bloom helicase (Blm), DNA polymerase λ (Pol λ), and Aprataxin and PNK-like factor (APLF). In addition, 7 of 10 NHEJ are present as ohnologs or duplicated ohnologs, well above the genome average of 40% [5]. Many NHEJ genes (DNAPKcs, Artemis, XRCC4, XLF, Ligase 4) were first described in mammalian V(D)J recombination and are absent in the model organisms *Drosophila* and *C. elegans*, and have therefore generally been considered “vertebrate specific.” However, homologs of DNAPKcs and Artemis were recently characterized in *Dictyostelium* [60] and have now been identified in automated annotation of multiple invertebrate genome assemblies.

#### Bloom helicase (Blm)

**Fig. 6:**
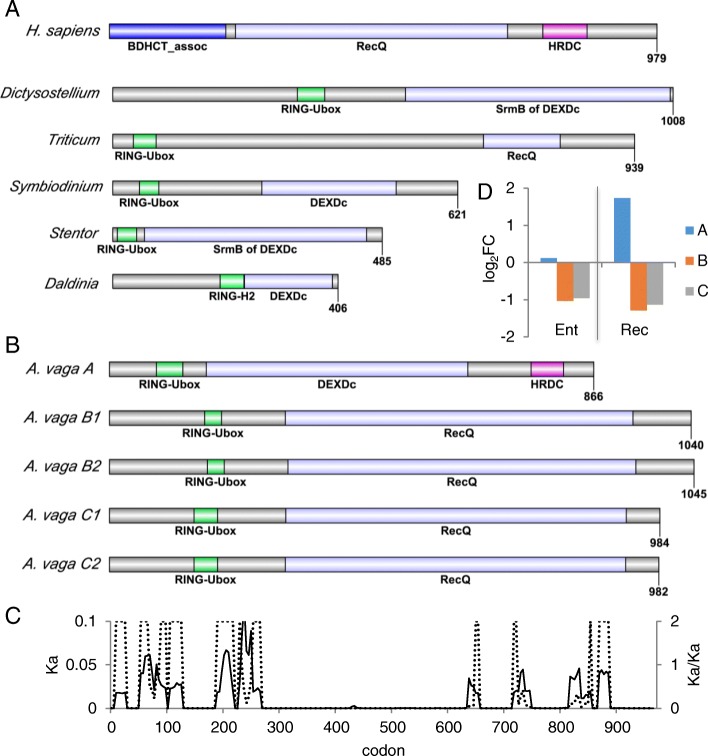
Bloom helicases. **a** Domain models of a Bloom helicase from *Homo sapiens* (XP_011520183) and Bloom-like helicases from *Dictyostelium purpureum* (XP_003287311.1, “hypothetical protein”), *Triticum monococcum* (AGH18689.1, “PHD-finger family protein”), *Symbiodinium microadriaticum* (OLP97093.1, “ATP-dependent RNA helicase DHH1”), *Stentor coeruleus* (OMJ79001.1, “hypothetical protein”), *Daldinia* sp. (OTB17292.1, “hypothetical protein”). **b** Domain models of the five copes of the Blm-like helicase from *A. vaga*. **c** Sliding window analysis of nonsynonymous (Ka) difference (solid line) and ratio of nonsynonymous to synonymous differences (Ka/Ks, dashed line) between copies C1 and C2. **d** Differential expression of *A. vaga* Blm ohnologs entering and recovering from desiccation, compared to hydrated controls. Values are log_2_ fold change of normalized counts, significance test values are listed in Additional file 1

*Adineta vaga* has five copies of a Bloom-like helicase: two allele pairs and a fifth copy on an assembly scaffold without an allele partner (Fig. 6). In humans, Blm is involved in many aspects of HR and also interacts with MMR proteins Mlh1 and Msh6 [61]. Human Blm contains a central RecQ domain flanked by an N-terminal BDHCT-box associated domain and a C-terminal Helicase and RNase Domain (HRDC); the BDHCT domain is weakly conserved in vertebrates and largely absent outside of chordates (Fig. 6a). Most metazoan Bloom-like proteins lack a BDHCT domain and many, such as the *Drosophila* homolog, also lack a HRDC domain but are active in homologous recombination [62]. In *A. vaga* all five copies have an N-terminal RING-Ubox superfamily domain; the two pairs (B1,2 and C1,2) have the canonical RecQ family helicase domain while the fifth copy (A) has only the more general DEAD-like helicase superfamily domain in this region (Fig. 6b). Only the A copy has an identifiable C-terminal HRDC domain. This domain appears to function as a protein to DNA binding bridge, and is critical for topoisomerase dissolution of Holliday junctions [63]. These Blm-like genes also present a rare case of potential functional divergence between allele copies: Copies C1 and C2 are 99% identical across the length of the protein, but only 95% identical in the first 270 aa. While the RING-Ubox domain is completely conserved, it is surrounded by regions where Ka/Ks are much greater than 1 Fig. 6c). The difference between the A copy and the B and C copies is reflected in profiles of differential transcript abundance entering or recovering from desiccation (Fig. 6d): expression of A increases more than 3-fold in recovery, while expression of B and C decrease about 2-fold both entering and recovering from desiccation.

#### Ku70/Ku80

**Fig. 7:**
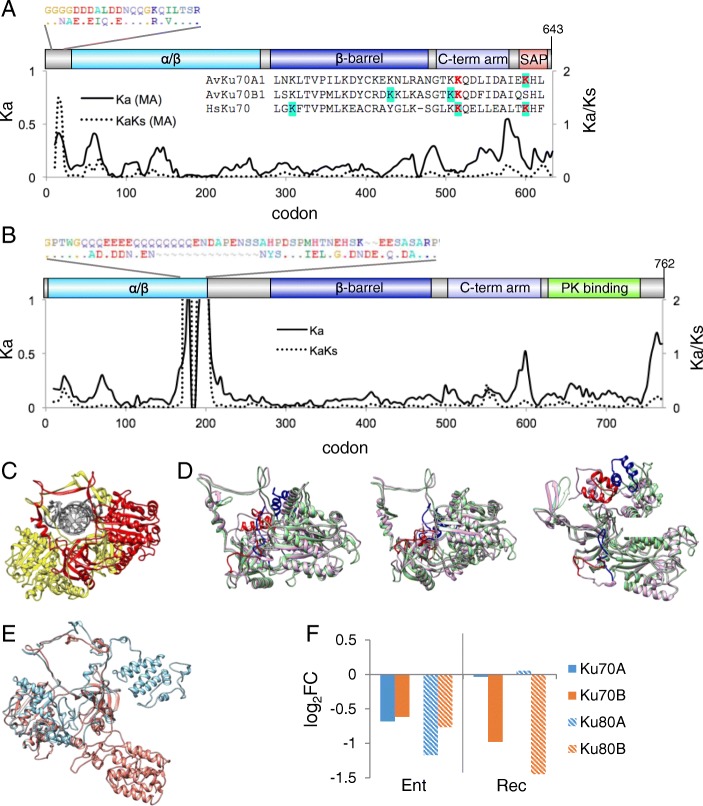
Ku70 and Ku80. **a** Domain model of *A. vaga* Ku70 A and B ohnologs, with sliding window analysis of nonsynonymous (Ka) difference (solid line) and ratio of nonsynonymous to synonymous differences (Ka/Ks, dashed line) between AvKu70A1 and B1. The alignment on the upper left shows the region where Ka/Ks > 1 near the N-terminus; the alignment to the lower right shows the SAP domains compared to human Ku70. Predicted sumoylation sites are in red, predicted acetylation sites are highlighted in blue. **b** Domain model of *A. vaga* Ku80 A and B ohnologs, with sliding window analysis of Ka and Ka/Ks. The alignment in the upper left shows the Q_3_E_4_Q_8_ track at the terminus of the α/β domain present in copy A and not in B. **c** Crystal structure PDB 1JEY, human Ku70 (yellow) Ku80 (red) heterodimer complexed with DNA (grey). **d** Three views of the superposition of the predicted structure of *A. vaga* Ku70A1 (purple) and *A. vaga* Ku70B1 (green). The N terminal region under putative positive selection and the SAP domain are indicated in red and blue for Ku70 A1 and B1, respectively. First orientation is the same as in (**c**), second is an elevated view (45° rotation along horizontal axis), third is a top view (90° rotation along horizontal axis). **e** Superposition of the predicted structures of *A. vaga* Ku80A1 (blue) and Ku80B1 (copper) in the same orientation as in (**c**). **f** Differential expression of *A. vaga* Ku70 A and B and Ku80 A and B ohnologs entering and recovering from desiccation, compared to hydrated controls. Values are log_2_ fold change of normalized counts, significance test values are listed in Additional file 1

Canonical NHEJ is initiated by the Ku70/Ku80 heterodimer threading onto exposed DNA ends. *Adineta vaga* has maintained ohnologous pairs of both Ku70 and of Ku80 and the conserved domains that characterize these proteins (Fig. 7). However, there is 20% amino acid difference between the ohnologs, and structural predictions suggests this divergence may affect function.

The Ku70 orthologs have similar structure through the N-terminal α/β domain and the central β barrel, but amino acid differences midway through the C-terminal arm cause divergent conformations that result in different positions of the respective SAP domains (Fig. 7a, c, d). The C-terminal arms of Ku70 and Ku80 embrace the β barrel of the opposite subunits [64] The SAP domain binds DNA and the affinity of Ku binding is modulated by sumoylation and acetylation of this region [65]. The A ohnolog has two predicted sumoylation sites in its SAP domain, while the B ohnolog has one; however the B ohnolog has an additional predicted acetylation site. There has been an accelerated rate of amino acid divergence at the N terminus (Fig 7a), a negatively charged disordered region that flanks the DNA accepting ring and may modulate Ku-DNA interaction [64].

Like Ku70, the Ku80 orthologs also have potentially different tertiary structure (Fig. 7b, c, e). While the beginning of the N-terminal α/β domain is conserved, including the APLF binding sites, there are several indels at the end of the domain, including a Q_3_E_4_Q_8_ track present only in the A ohnolog. Amino acid differences at the end of the central β barrel and beyond predicted different orientations of both the C-terminal arm and DNAPKcs interaction region. There has been an accelerated rate of amino acid divergence at the C terminus, a region that interacts with DNAPKcs to result in different DNAPKcs activity depending on whether the DNA end has a 5′ overhang, a 3′ overhang, or is blunt [66].

All copies of Ku decreased in expression entering desiccation, but upon recovery Ku70A and Ku80A rebounded quickly to the expression level seen in the hydrated controls (Fig. 7f).

#### DNAPKcs

**Fig. 8:**
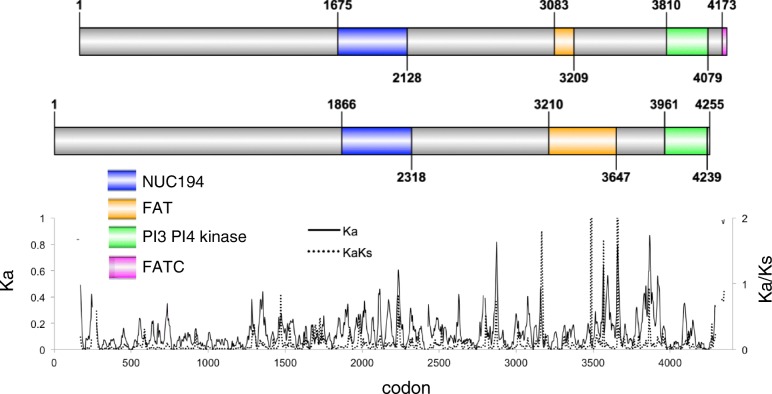
DNAPKcs. Domain model of *A. vaga* DNAPKcs A (top) and DNAPKcs B (bottom) ohnologs with sliding window analysis of nonsynonymous difference (Ka, solid line) and ratio of nonsynonymous to synonymous differences (Ka/Ks, dashed line) between A1 and B1

*Adineta vaga* has two orthologs of DNAPKcs (Fig. 8), a large DNA-dependent serine/threonine protein kinase that forms a ring structure that combines with Ku70 and Ku80 at either side of a DSB [67, 68]. The A ohnolog is 200 aa shorter than the B ohnolog in the N-terminal region, which builds the ring structure. The two ohnologs also differ in the presence of conserved domains: the A ohnolog has a NUC194 domain, a degenerated FAT domain, a phosphatidylinositol 3- and 4- kinase (PI3_PI4_kinase) domain, and an FATC domain. The B ohnolog shares the NUC194 and PI3_PI4_kinase domains but has an intact FAT domain and lacks the C terminal FATC domain. Although human DNAPKcs possesses all of these domains, the DNAPKcs of most eukaryotes do not. The domain structure of the A ohnolog (lacking an intact FAT domain) is also found in protists and fungi, while the domain structure of the B ohnolog (lacking the FATC domain) is found in some lophotrochozoans and in some mammals. Examples of both structures can be found in arthropods.

DNAPKcs undergoes conformational changes after DNA-binding and extensive autophosphorylation that in turn influence its kinase and repair activities [67]. The ohnologs share 45 predicted autophosphorylation sites, including three positions where one ohnolog has a serine and the other a threonine. However, there are 34 predicted autophosphorylation sites in DNAPKcs copy A that are not in B and 26 predicted autophosphorylation sites in B that are not in A. Sliding window analysis of Ka/Ks along an alignment of the ohnologs reveals several regions of increased amino acid divergence, including the region where the FAT domain has degenerated in the A type. Transcript abundance of both ohnologs decreases during entry and recovery from desiccation (not shown).

#### Artemis

**Fig. 9:**
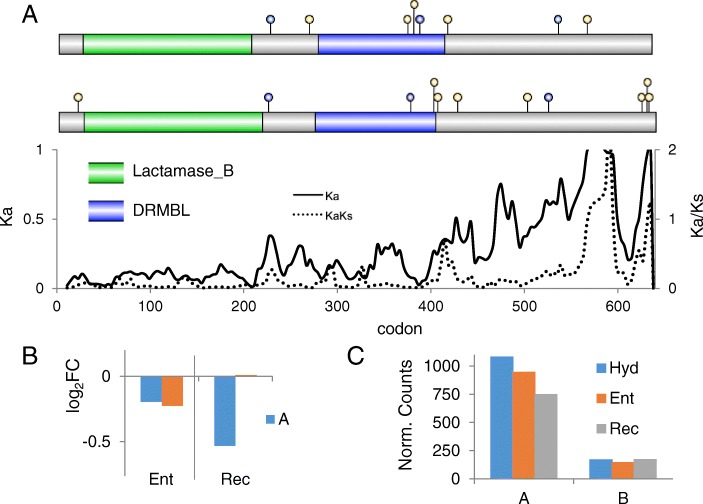
Artemis. **a** Domain models of *A. vaga* Artemis A (top) and B (bottom) ohnologs showing predicted DNAPKcs phosphorylation sites (blue circles indicate sites conserved between A and B peptides, yellow circles indicate unique sites on each peptide) with sliding window analysis of nonsynonymous difference (Ka, solid line) and ratio of nonsynonymous to synonymous differences (Ka/Ks, dashed line) between A1 and B1. **b** Differential expression of *A. vaga* Artemis A and B ohnologs entering and recovering from desiccation, compared to hydrated controls. Values are log_2_ fold change of normalized counts, significance test values are listed in Additional file 1. **c** Normalized read counts of the two ohnologs under hydrated, entering, and recovering conditions

In addition to direct action by the DNAPKcs-Ku70-Ku80 holoenzyme, accessory nucleases and phosphorylases are recruited to prepare broken DNA ends for ligation. Chief among these is Artemis, which acquires endonuclease activity upon phosphorylation by DNAPKcs to produce blunt ends from a wide variety of DNA structures [69]. The two ohnologs of Artemis in *A. vaga* are conserved (93% amino acid identity) over the first two thirds of the protein (Fig. 9a), which contains the catalytic metallo-beta-lactamase (Lactamase_B) and DNA repair metallo-beta-lactamase (DRMBL) domains. However, there are differences in the predicted number and position of sites that would be phosphorylated by DNAPKcs, and the C terminal regions share only 45% amino acid identity. This region is poorly conserved across animals, but physical interactions with the N terminal portion are believed to regulate nucleolytic activity through autoinhibition [70]. The transcript abundance of the A ohnolog is significantly decreased during recovery from desiccation while the B ohnolog is maintained at hydrated levels entering and recovering from desiccation (Fig. 9b). In addition, expression of the A ohnolog is 4–5 times more abundant than the B ohnolog in all conditions (Fig. 9c).

#### XRCC4

**Fig. 10:**
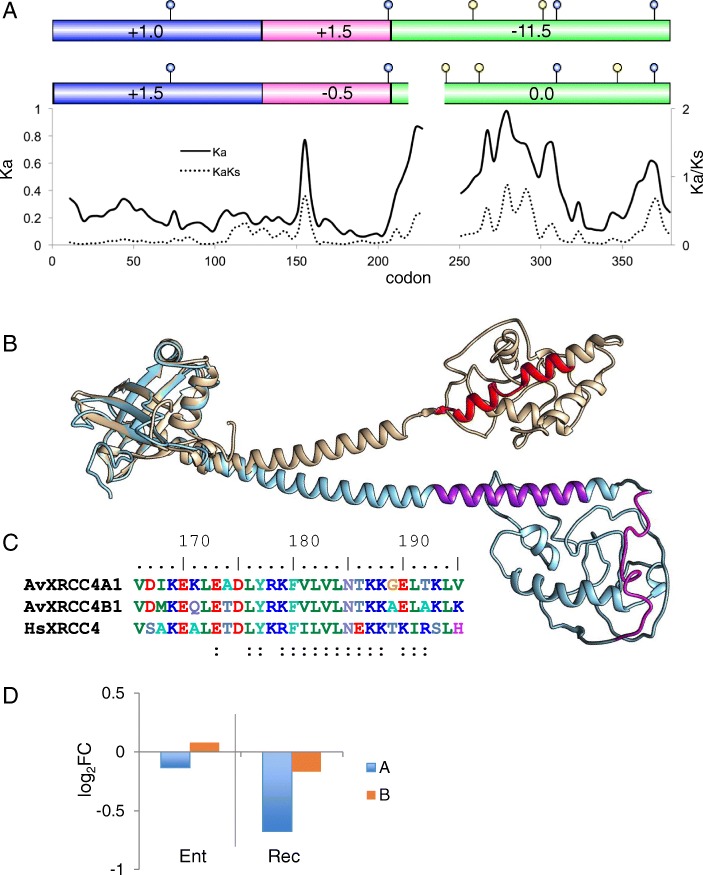
XRCC4. **a** Domain models of AvXRCC4 A (top) and B (bottom) ohnologs showing the three regions of XRCC4, the total charge of each region, and predicted DNAPKcs phosphorylation sites (blue circles indicate sites conserved between A and B peptides, yellow circles indicate unique sites on each peptide) with sliding window analysis of nonsynonymous difference (Ka, solid line) and ratio of nonsynonymous to synonymous differences (Ka/Ks, dashed line) between copies A1 and B1. **b** Superposition of the predicted structures of *A. vaga* XRCC4A1 (blue) and XRCC4B1 (copper) showing the conserved structure of the N-terminal head region, the central helix with Ligase 4 binding regions shown in purple (XRCC4A1) and red (XRCC4B1), and the poorly conserved C terminus with 22 residues present in A1 but not in B1 shown in magenta. **c** Alignment of the Ligase 4 binding region in A1, B1, and human XRCC4; colons (:) indicate residues involved in binding [116]. **d** Differential expression of *A. vaga* XRCC4 A and B ohnologs entering and recovering from desiccation, compared to hydrated controls. Values are log_2_ fold change of normalized counts, significance test values are listed in Additional file 1

At the conclusion of NHEJ, ligation of DNA ends is achieved by the XRCC4-XLF-Lig4 holoenzyme. XRCC4 forms a homodimer that acts as a scaffold, interacting with Ku, DNAPKcs, XLF, Lig4, and the DNA. It is composed of an N terminal beta-barrel that interacts with XLF and other peptides, a ~ 80 aa helix that tightly binds Lig4 and interacts with DNA, and a poorly characterized, largely disordered C terminal domain that may contact the N terminal domain to stabilize protein interactions [71, 72]. The ohnologs of XRCC4 in *A. vaga* are more divergent (60% identity) than any other ohnologs in NHEJ (Fig. 10a). While the Ligase 4 binding domain is conserved, two kinks in the helix domain of the B ohnolog may affect Ligase 4 binding, DNA association, or both (Fig. 10b,c). The greatest difference between ohnologs occurs in the C terminal domain, with only 46% amino acid identity and several indels accounting for a difference in length of 34 amino acids, or nearly 20% of the region. Amino acid differences and indels result in a different pattern of predicted DNAPKcs phosphorylation sites and a substantial charge difference in the region, with net charges of − 11.5 and 0 for the A and B ohnologs, respectively. The transcript abundance of the A ohnolog is significantly decreased during recovery from desiccation while the transcript levels of the B ohnolog are maintained at hydrated levels entering and recovering from desiccation (Fig. 10d).

#### DNA polymerase λ

Both Polβ and Polλ are present in *A. vaga*, notable in part because both X-family polymerases were until recently thought to be absent from protostomes [73, 74]. There are six Polλ copies (three pairs) in *A. vaga* while every other replicative, repair, and TLS polymerase lacks ohnologs. Polλ is the primary gap-filling polymerase of NHEJ, and can participate in BER along with Polβ, the primary gap-filling polymerase of that pathway [75, 76]. Both Polβ and Polλ can also perform TLS [77], but they are not generally categorized as TLS polymerases.

**Fig. 11:**
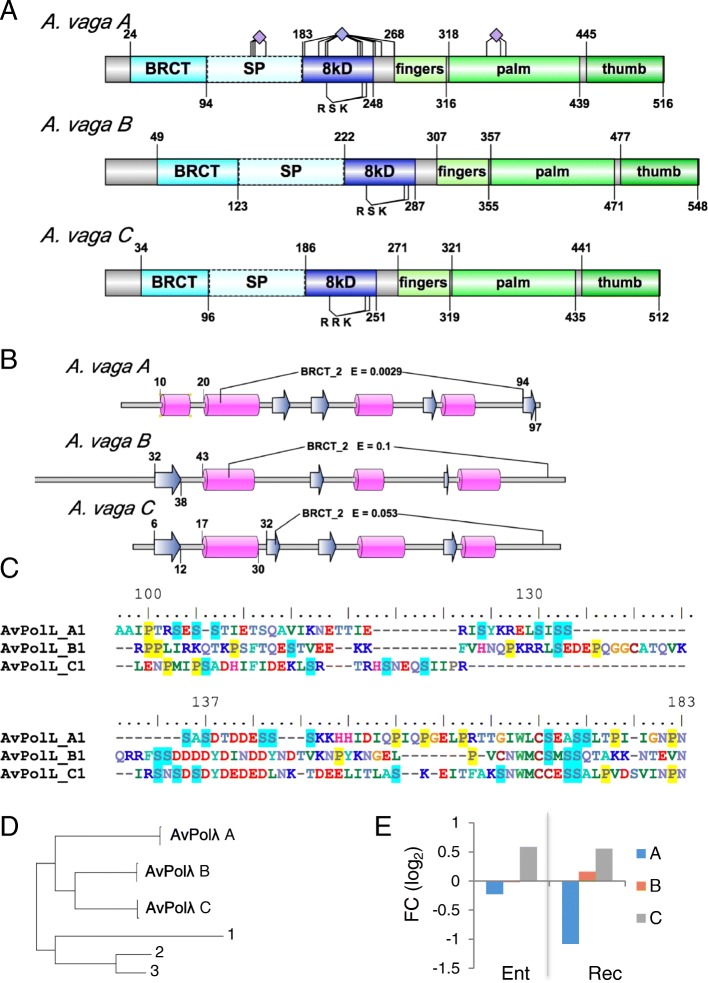
Ohnologs of Polλ. **a** Domain structure of the three types of polymerase λ in *A. vaga*. Boundaries of domains defined by hmmscan of PfamA are shown above (start) and below (end); the disordered Ser/Pro-rich region (SP) is not a defined domain. The three residues that make up the phosphate binding pocket in the 8kD domain are shown (RRK or RSK). The position of residues encoded by codons determined to be under positive selection in the lineage leading to AvPolA are shown above the *A. vaga* A structure clustered by diamonds for each domain. **b** Secondary structure of the BRCT domain in polymerase λ as determined by Phyre. Beta sheets are shown as blue arrows, alpha helices as pink cylinders. The region identified as the Pfam domain BRCT_2 by hmmscan is shown with domain-specific expectation value; positions of structure boundaries outside of the predicted BRCT domain are indicated. **c** Alignment of the disordered SP region in *A.* vaga copies of polymerase λ. Numbering is to AvPolLA1. Serine and proline residues are highlighted in blue and yellow, respectively. **d** Unrooted gene tree of the six copies of Pol λ in *A. vaga* and three additional rotifer species used for codeml tests of selection. 1, *Seison* sp.; 2, *Brachionus manjavacas*; 3, *Brachionus calyciflorus*. **e** Differential expression of the three paralogs entering and recovering from desiccation, compared to hydrated controls. Values are log_2_ fold change of normalized counts, significance test values are listed in Additional file 1

Both Polβ and Polλ have 5′-deoxyribose-5-phosphate lyase domains to remove chemical groups that would block ligation. Polλ interacts with NHEJ proteins through its BRCT domain, while the serine-proline-rich region affects fidelity and may be post-translationally modified [78, 79]. The three pairs of Polλ in *A. vaga* all have the same domains but vary in length, and the relative location of the BRCT domain shifts between the copies, spanning different secondary structures, which themselves are not entirely conserved (Fig. 11a,b). Only the C pair shares the human Polλ secondary structures, but it also has the smallest BRCT domain. There is substantial sequence divergence between the copies in the serine-proline rich regions (Fig. 11c). Finally, we find evidence that multiple sites within the 8kD lyase domain, the flanking regions, and the palm domain are under positive selection in the A pair.

Although all copies appear capable of producing properly spliced coding sequence, one of the A copies and one of the C copies are not expressed during any condition of the RNA-Seq experiment. Transcript abundance of the expressed copy A is significantly decreased during recovery from desiccation, while the transcript abundance of the B copies remains unchanged entering or recovering from desiccation (Fig. 11e). Transcript abundance of the expressed C copy is significantly higher entering and recovering from desiccation.

#### APLF

**Fig. 12:**
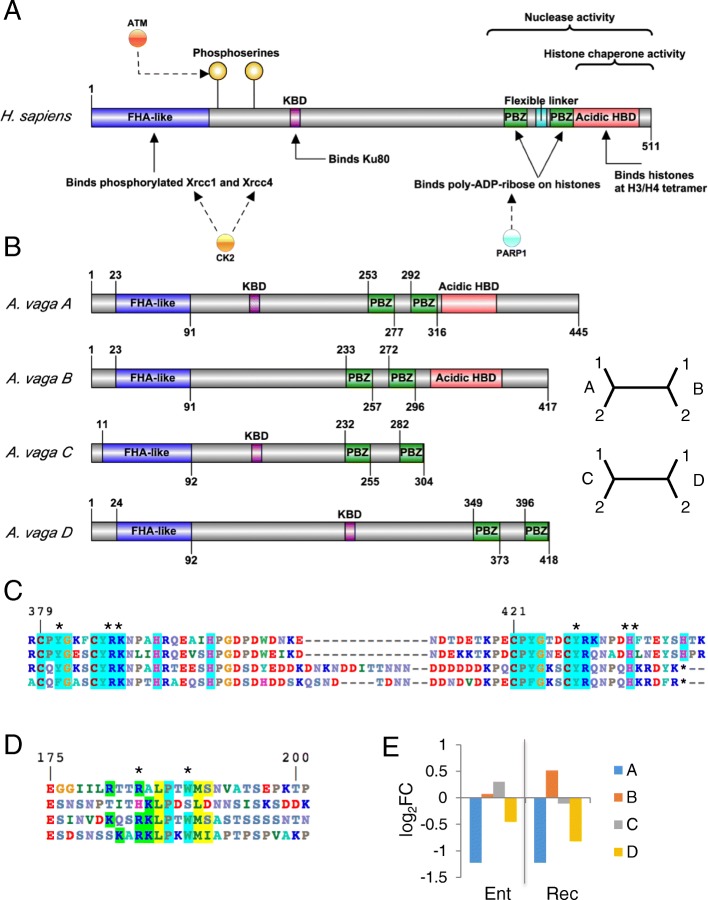
Ohnologs of APLF. **a** Domains and interactions of *H. sapiens* APLF assigned using Uniprot Q81W19 as template with domain annotation and function refined with reference to [60,83,84,93,119]. **b** Domain models and phylogenetic relationship of the two pairs of *A. vaga* APLF ohnologs, with cladograms showing the relationship of gene copies and ohnologs. **c** Alignment of the tandem PBZ domains. Each PBZ domain has a conserved C(M/P)Y and CRY motif, highlighted in aqua along with nearby conserved residues, and these form a basic, hydrophobic pocket for ADP-ribose binding. APLF binds multiple ADP-ribose residues within PAR, and Y381/Y386 and Y423/Y428 are critical for interactions with the adenine ring, and R387/R429 coordinate the interactions with the phosphates [91]. All are marked with (*). The Y423F difference in D is found in some other metazoans. The C and D ohnologs substitute Q for P in the first PBZ domain, which would not be expected to maintain the characteristics of the basic, hydrophobic binding pocket. Both also terminate before the final H of the second PBZ domain, which undoubtedly alters the domain’s binding properties. **d** Alignment of the Ku80 Binding Domain (KBD) regions. Copies A, C and D retain R184 and W189, the residues found critical for Ku binding in mammals [117]; copy A lacks one of the conserved positively charged residues found in most KBD domains. **e** Differential expression of all four paralogs entering and recovering from desiccation, compared to hydrated controls. Values are log_2_ fold change of normalized counts, significance test values are listed in Additional file 1

APLF is an intrinsically disordered protein that participates in both BER and NHEJ. Typically identified as an accessory in NHEJ, APLF has remarkable spatial and temporal spans, interacting with core NHEJ members at the beginning and end of the break repair process and participating in multiprotein assemblages of up to six proteins. APLF has two DNA-damage dependent phosphorylation sites, a Ku80-binding domain, two poly(ADP-ribose) (PAR)-binding zinc fingers (PBZ) associated with both histone interactions and nuclease activity, and an acidic region with histone chaperone activity [58, 59, 71, 80–82] (Fig. 12a). APLF is able to recognize and incise at abasic sites and at some damaged sites, including hydroxyuracil, hydroxycytosine and thymine glycol [58]. Remarkably, *A. vaga* has two sets of ohnologs of APLF (A:B and C:D), each with a different domain structure (Fig. 12b). The C and D ohnologs do not have the C-terminal histone-binding and chaperone region**,** the loss of which effectively places their non-canonical PBZ domains at the C-terminal end of the protein (Fig. 12b,c). The Ku80 binding domain is well conserved in the C and D ohnologs, less conserved in the A ohnologs, and poorly conserved in the B ohnologs (Fig.12d). Each ohnolog also has a distinct expression pattern during desiccation: abundance of ohnolog A transcripts decrease during entry and recovery, ohnolog B transcripts increase during recovery, ohnolog C transcripts are unchanged in desiccation, and ohnolog D transcripts decrease during recovery (Fig. 12e).

## Discussion

Bdelloids have evolved under selective pressure to cope with desiccation and its damaging effects to DNA. We found numerous DDR genes retained in *A. vaga* that have been lost in classic invertebrate models, genes incorporated by horizontal transfer, and genes present in unexpectedly high copy numbers. These patterns were non-randomly distributed in DDR: with the exception of Blm, all were in either BER or NHEJ, the two main pathways that repair oxidative damage. Within this collection of genes several themes emerge: resilience through increased damage recognition and end repair capability; modifications in DSB repair that may promote gene conversion between divergent sequences; and the potential functional divergence between ohnologs.

### Damage recognition and end repair

Several *A. vaga* excision repair glycosylases and endonucleases, including three of non-metazoan origin (Fpg, UVDE, and AlkD), recognize multiple substrates in other systems. UVDE and AlkD can excise both small base lesions usually repaired by BER or MMR, and bulky lesions repaired by NER [47, 55]. UVDE and APLF can initiate AER by producing a single nick at abasic sites and at certain damaged bases [37]. The horizontally-acquired Fpg of *A. vaga* may also act as an endonuclease to initiate AER if, like homologs from *Candida albicans* and *A. thaliana*, its preferred substrate is an abasic site [42]. That APLF, the DDR gene present in the most copies, and three of the four horizontally-acquired DDR genes are all involved in damage recognition and removal suggests to us that enhancing those functions can improve resilience to oxidative damage.

End repair to remove chemical blockages preventing synthesis or ligation is rate-limiting in BER and in NHEJ [83, 84]. A phosphate, unsaturated sugar residue, or other non-hydroxyl group at the 3′ end of DNA is a block to repair synthesis by a DNA polymerase; a 5′ blocking group, often a 5′-deoxyribosephosphate (5-dRP), will prevent ligation. Artemis and APLF can remove 3′ synthesis blocking groups, while Artemis, Polλ, and the Ku heterodimer have 5’-dRP lyase activity. The shared PBZ domains of APLF and LigK suggest that LigK may have a 3′-end processing activity [58, 59]; LigK could also have a 5′ end processing function to remove blocks to ligation similar to some bacterial NHEJ ligases [85, 86]. These results suggest that end-repair genes have been incorporated into DDR by horizontal transfer or duplication because they improve rate limiting steps, augmenting end repair to improve resilience to oxidative damage.

### DSB repair and gene conversion

Bdelloid oocytes are arrested at G_0_ [87], a condition which would favor DSB repair by NHEJ over HR due to the lack of sister chromatids. The proliferation of NHEJ genes compared to HR genes in *A. vaga* is consistent with a prominent role of NHEJ in bdelloid DDR. However, a role for HR in *A. vaga* is suggested by evidence of extensive gene conversion between former alleles that are much more divergent than sister chromatids [5, 8]. Higher rates of conversion between divergent sequences may be moderated by the absence of Rad52, BRCA2, and MMR genes, and possibly by the abundance of Blm helicases [88, 89]. Blm has roles promoting synthesis-dependent strand annealing-type HR and in dissolving double-Holliday junctions, both of which convert genes between homeologous sequences [88, 90]. We and others have hypothesized that gene conversion between divergent gene copies in bdelloid mimics meiotic recombination by exposing recessive alleles to selection, potentially allowing and escape from Muller’s Ratchet [5, 8, 19].

### Functional divergence of multiple copy genes

We found that the multiple copies of LigK and the majority of NHEJ genes (Ku70, Ku80, DNAPKcs, Artemis, XRCC4, APLF, and Polλ) have regions of high primary sequence difference, differences in predicted modifications such as phosphorylation, differences in predicted secondary or tertiary structure, differences in expression pattern through the desiccation process, or a combination of these. In the case of LigK, the different number of PBZ domains is likely to confer very different functional properties to the two ohnologs. Studies of the PBZ domains of human APLF suggest that tandem PBZ domains act cooperatively, with a binding affinity to poly(ADP-ribose) 1000 times greater than single domains [91]. Unlike the A ohnolog with a single PBZ domain, the B ohnolog with two PBZ domains is significantly overexpressed during recovery from desiccation.

While desiccation may be only one driver of functional divergence between ohnologs, and transcript abundance profiles are an imprecise measure of activity, it is remarkable that seven of the ten NHEJ genes we examined have ohnologs and that six of these show different expression profiles between ohnologs in desiccation. The expression profiles of Ku70 and Ku80 A and B ohnologs suggest specific pairing of these heterodimers. DNAPKcs is remarkable for having ohnologs that have different domain structures that have each evolved independently multiple times in different eukaryotic lineages. In the case of APLF, differences in the Ku binding domain between ohnologs may alter both Ku80-binding and XRCC1/4 binding by the nearby FHA domain, and the absence of the C-terminal histone binding region in C and D orthologs and non-canonical residues within their PBZ domains suggest they would bind PAR very differently than the A and B orthologs. Length differences between the short, compact structure of the C ohnolog versus the extended D ohnolog likely cause differences in scaffolding ability. The three ohnologs of Polλ show reduced expression, no change, and increased expression in response to desiccation. The proteins differ in a number of structural features and coding sequence show evidence of positive selection between copies. At this time, Polλ is the only DDR gene for which there are sequences available from enough non-bdelloid rotifers to perform these tests for selection. The four ohnologs of APLF also each have a different expression profiles.

The large number of NHEJ genes maintained in multiple copies is even more remarkable considering that when these genes are present in species other than bdelloid rotifers, they are invariably found only in single copies (the only exception we have identified are recent duplications of Ku70 and/or Ku80 in some fish genera). This suggests that other species are unable to maintain duplications of these genes as they inevitably diverge through mutation accumulation [92].

## Conclusions

A unifying feature of all of the genes for which we find evidence of functional divergence is that they each have multiple roles in DNA repair. If these disparate functions cannot simultaneously be optimized by selection in a single copy of the gene, duplication would allow an escape from this adaptive conflict through subfunctionalization and potential eventual neofunctionalization [93]. This raises the question of why, if the advantage is so clear, other animals have not retained duplicated copies of these genes. We suggest that given the complexity of these proteins and their importance to fitness it is difficult for duplicates to evolve neutrally as generally envisioned in models of subfunctionalization [94]. If gene duplicates accumulate mildly deleterious mutations, sexual recombination will quickly eliminate them from a population before new beneficial structures can evolve. Because asexual species are much less efficient at removing deleterious mutations, it may be possible for them to cross fitness landscapes that sexual species cannot. This would suggest a potential long-term advantage to asexuality that could help explain both the large scale retention of duplicated genes in bdelloids and their evolutionary success.

## Methods

### Identification, annotation and analysis of DNA repair genes

We annotated the gene calls from the *Adineta vaga* genome assembly [5] available from http://www.genoscope.cns.fr/adineta/data/ or ftp://ftp.ensemblgenomes.org/pub/metazoa/release-41/fasta/adineta_vaga/dna/ using protein BLAST [95, 96] to KEGG [97] and NCBI refseq databases [98], considering evalue, aligned length, percent identity, bit scores, and reciprocal best BLAST confirmation. About one third of the identified genes appeared to have an odd number of copies, to have short coding sequences relative to alleles and/or ohnologs, or to be fragmented into multiple consecutive gene calls. We edited these using Genewise [99], manual curation, and RNA-Seq mapping results (described below), resulting in new or improved annotations for two-thirds of the inspected genes. We designate ohnologs as A, B, etc. and allele copies as A1, A2 or B1, B2 following [9].

We used the Phyre2 server [100] to predict protein secondary and tertiary structure and Chimera 1.11.2 [101] to visualize and compare models. Chimera is developed by the Resource for Biocomputing, Visualization, and Informatics at the University of California, San Francisco (supported by NIGMS P41-GM103311). We identified protein functional domains using hmmsearch and hmmscan tools in HMMER v3.1 [102] to Pfam-A v30.0 [103], or the NCBI CD-Search tool [104] to the NCBI Conserved Domain Database [105]. We used Illustrator for Biological Sequences [106] to visualize protein domain architecture. We used the Expresso mode of T-Coffee Version_11.00.8cbe486 [107, 108] to align predicted amino acid translations of *A. vaga* gene copies with orthologs and other homologs identified by BLASTP searches of NCBI databases.

We generated gene trees using RAxML 8.2.11 [109] and MrBayes v3.2.6 [110]. For RAxML, we chose the amino acid model with the best likelihood using the PROTGAMMAAUTO option, and searched for the best maximum likelihood tree with bootstrap support from 1000 replicates. For MrBayes, we estimated the appropriate evolutionary model (“prset aamodelpr = mixed”) and compared two runs of 4 chains each after 4 × 10^6^ generations, sampling every 100 generations and discarding the first 30,000 samples as burn-in. Convergence diagnostics ESS, PSRF, and the average standard deviation of the split frequencies between runs were always > 100, 1.000–1.010, and < 0.10, respectively. To guard against erroneous placement of *A. vaga* sequences due to long-branch attraction effects we used the posterior Bayes factor approach of [111] as implemented in pbf v1.0 to adjust the posterior probability of each tree and split and examined the placement of *A. vaga* sequences relative to other clades.

For a list of all species and accession numbers used for each gene, RAxML command lines and MrBayes blocks, and complete maximum likelihood and Bayesian trees with bootstrap or posterior probability support, see Additional file 2.

We used Ka_Ks_Calculator 2.0 on sliding windows of coding sequence alignments to determine Ks and Ka using the Model Averaging method [112]. Where we had constructed multiple alignments for phylogenetic analysis, we extracted aligned sequence pairs from the alignments described above and removed shared gaps; otherwise we aligned peptide pairs using Expresso as described above. We conducted both site and branch–site tests of positive selection using codeml in PAML v4.9c. Non-default parameters in our codeml control file for site tests were CodonFreq = 1, model = 0, NSites = 0 1 2 7 8, omega = 0.4, and we used the likelihood ratio test to compare NSites = 2 vs NSites = 1 with df = 2 and NSites = 8 and NSites = 7 with df = 1. For branch-site tests we changed to model = 2, NSites = 2 and tested fix_omega = 0, omega = 1.5 and omega = 1, fix_omega = 1.

### Rotifer desiccation, recovery, and collection

We collected 3 biological replicates each of rotifers entering desiccation, recovering after 7 days of desiccation, and from hydrated controls. Our culture of *A. vaga* is derived from a single egg and grown in 6-well tissue culture plates in filtered spring water on a diet of *E. coli*, as previously described [9]. For each replicate, we pooled nine 6-well dishes of *A. vaga*, added approximately equal number of animals to each of nine 150-mm tissue culture plates coated with 3% low-melting point agarose. A serological pipet with its tip wrapped in 10-um nylon mesh was used to remove 25-mL liquid from each plate. The plates were set bench-top at ambient temperature (~ 21 °C) and humidity with their lids raised ~ 0.5 cm on three paperclips attached to the sides of the lower dish. Plates were observed daily. Hydrated controls were collected after 2 days. Animals entering desiccation were collected after 4–6 days, when a water film remained and animals were often contracted, but still showed some movement. Desiccation started at or near day 10, when the agarose was dry and the rotifers had contracted into tuns. After 7 days in desiccation, 10 mL (final post-agarose-rehydration volume) sterile spring water was added to each plate. The rotifers began moving almost immediately and were harvested after 1 h of recovery by adding 20-ml HBSS to each dish and transferring rotifers, water, and buffer to sterile 35-mL centrifuge tubes on ice. The rotifers were pelleted 5 min at 5000 rcf at 4 °C, then all but 1-mL of supernatants were removed; rotifers were resuspended in the remaining liquid and transferred to 1.5-mL tubes on ice. Rotifers were then pelleted 1 min at 2000 rcf, room temperature, followed by complete removal of supernatant. Pellets were directly processed as described below. Culture dishes, serological pipets, and Autofil 0.22um PES filter bottle assemblies were from USA Scientific, Ocala, FL. Poland Spring water (Poland Spring, ME) is used for cultures. Gridded, 150-mm tissue culture plates were from Falcon Corning, Corning, NY. Low-melting point agarose was from Fisher Scientific, Pittsburgh, PA; and nylon 10-um Nitex mesh was from Sefar America, Inc., Kansas City, MO. Hank’s Buffered Saline Solution (HBSS) without calcium, magnesium or phenol red was from Thermo Fisher Scientific/ Gibco-Life Technologies, Waltham, MA.

### RNA-Seq library construction and sequencing

For each replicate, RNA was extracted from 20-mg *A. vaga* pellets collected from hydrated rotifers, rotifers entering desiccation, and rotifers recovering from desiccation, using TRIzol Reagent per the manufacturer’s protocol, with the addition of linear acrylamide as a co-precipitant. An additional extraction with RNeasy MinElute prior to DNA removal by TURBO DNA-free Kit removed agarose carryover. RNA was quantified with RiboGreen and qualified by 260/230 ratio and Bioanalyzer RNA 6000 Pico Kit. Libraries were constructed using the KAPA Stranded mRNA-Seq Kit with Illumina TruSeq indexed adapters from 625 ng total RNA per sample. Magnetic bead binding steps in the library preparation protocol were performed in 1.5-mL tubes. Final libraries were assessed with PicoGreen and Bioanalyzer High-Sensitivity DNA chip prior to pooling and size selecting 445 bp using a Pippin Prep 1.5% agarose gel cassette. Final quantification of amplifiable library fragments was done by qPCR. Libraries were sequenced on three separate Illumina NextSeq runs with 150 bp paired-end reads and a dedicated index read. An average of 27,000,000 paired-end, 150-bp reads (over 7Gb) were obtained per library and 91% passed quality filtering [113]. Reads are available from the NCBI Short Read Archive under accessions SRR7962065–SRR7962073 or as BioProject PRJNA494578. TRIzol Reagent, TURBO DNA-free Kit, Quant-iT RiboGreen RNA Assay, and Quant-iT PicoGreen dsDNA Assay Kit were from Thermo Fisher Scientific. Linear acrylamide was from AMRESCO, Solon, OH and the RNeasy MinElute Cleanup Kit was from Qiagen, Germantown, MD. RNA 6000 Pico and High-Sensitivity DNA kits were from Agilent Technologies, Santa Clara, CA. KAPA Stranded mRNA-Seq Kit and Library Quantification (qPCR) Kit for Illumina platforms were from Kapa Biosystems, Wilmington, MA. Illumina TruSeq indexed adapters were from Integrated DNA Technologies (IDT), Coralville, IA. Pippin Prep 1.5% agarose gel cassettes were from Sage Science, Beverly, MA.

### RNA-Seq analysis

To aid gene annotation, results from two of the replicate hydrated libraries were mapped to the annotated *A. vaga* genome using the RNA-Seq Analysis tool in CLC Genomics Workbench 8.5.1. Mapping was done to genes plus intergenic regions with the following parameters: 80% length, 90% similarity, mismatch cost = 2, insertion cost = 3, deletion cost = 3, no global alignment, reverse strand specific, auto-detect paired distances, maximum of 10 hits per read, count paired reads as two, and expression value = total counts. Final transcript validation was performed by mapping results from all nine libraries to the genome using the Large Gap Mapping tool in CLC Genomics Workbench 10.0.1 with the following parameters: max number hits per segment = 6, max distance from seed = 2000, multi-match mode = random, mismatch cost = 2, insertion cost = 3, deletion cost = 3, similarity = 0.96, length fraction = 0.9, override default distances = no.

After complete curation of DDR genes, each library was mapped using RSEM 1.3.0 [114] to the complete *A. vaga* transcriptome reference edited by replacing 58 DDR coding sequences with improved annotation as describe above. Differential expression was assessed using EBSeq 1.2.0 [115] invoked from the rsem-run-ebseq script in RSEM using default values; and DESeq2 v1.10.1, EdgeR v3.12.1, and limma v3.26.9 invoked from the run_DE_analysis.pl script in Trinity v2.2.0 using default values. Significant differential expression (SDE) was PPDE > 0.95 for EBSeq, padj < 0.05 for DESeq, or fdr < 0.05 for edgeR and limma-voom. The statistics underlying padj, PPDE, and fdr are different and an arbitrary alpha = 0.05 is not equivalent across methods. A bash script detailing all program invocations is available as Additional file 3.

We compared the reproducibility of our replicate libraries by examining scatterplots of transcript mapping reported as transcripts per million (TPM). R^2^ values were > 0.99; 0.92–0.97; and 0.88–0.99 for the hydrated, entering, and recovering replicates, respectively. The greater variance in entering and recovering libraries were each due to single libraries. To assess whether these libraries affected the geometric mean size factor used to normalize read counts in EBSeq and DESeq we examined the distribution of the ratio of the counts per gene and the geometric mean for each library, and in all cases found a unimodal histogram with the size factor at or near the mode. Repeating the differential expression analyses without these libraries had no significant effect on significant differential expression of DDR genes.

## Additional files

Additional file 1: All Examined DNA Damage Response Genes. This Excel file is a table containing all gene names, A. vaga genome accession numbers, genome coordinates, expression levels, and results of differential expression significance tests. (XLSX 132 kb)

Additional file 2: Details of Phylogenetic Analyses. This pdf contains scripts for RAxML and MrBayes analyses, complete gene trees and tables genes used for each tree (OTU designation, Accession, and species name). (PDF 692 kb)

Additional file 3: Script for RNASeq Analysis. This Word file contains a bash script to run the rsem mapping and differential gene expression tests. (DOCX 113 kb)

## Abbreviations

AER: Alternate Excision Repair
BER: Base Excision Repair
DDR: DNA damage response
DSB: Double-strand break
MMR: Mismatch Repair
NER: Nucleotide Excision Repair
NHEJ: Non-Homologous End Joining
SSB: single strand break
TLS: translesion synthesis
