# Supplementary material for: Evolutionary diversity and novelty of DNA repair genes in asexual Bdelloid rotifers
Source: BMC Evol Biol. 2018 Nov 28;18:177. doi: 10.1186/s12862-018-1288-9 (PMC6264785; doi:10.1186/s12862-018-1288-9)
Supplement: Supplementary file 2 — Details of Phylogenetic Analyses. This pdf contains scripts for RAxML and MrBayes analyses, complete gene trees and tables genes used for each tree (OTU designation, Accession, and species name). (PDF 692 kb) [file 12862_2018_1288_MOESM2_ESM.pdf]

Additional File 2

This file contains scripts for RAxML and MrBayes analyses, complete gene trees and tables genes used for each tree (OTU designation, Accession, and species name)

```
# RAxML
PROGRAM= raxml-8.2.11/raxmlHPC-PTHREADS
# determine the best model for each alignment
$PROGRAM -T 4 -m PROTGAMMAAUTO -p $RANDOM -s $INFILE -n $OUTFILE
# check number of partitions for number of threads to use in next step
#bootstrap plus ml tree, bs values on best tree
$PROGRAM -T $THREADS -f a -x $RANDOM -p $RANDOM -N 1000 -m PROTGAMMAWAGF \
  -s $INFILE -n $OUTFILE
```

```
[MrBayes 3.2.6]
begin mrbayes;
set autoclose=yes nowarnings=yes;
prset aamodelpr=mixed;
mcmcp relburnin=no ngen=4000000 printfreq=1000 samplefreq=100 nchains=4
savebrlens=yes;
mcmc append=yes;
sump relburnin=no burnin=30000;
sumt relburnin=no burnin=30000 contype=allcompat;
quit;
end;
```

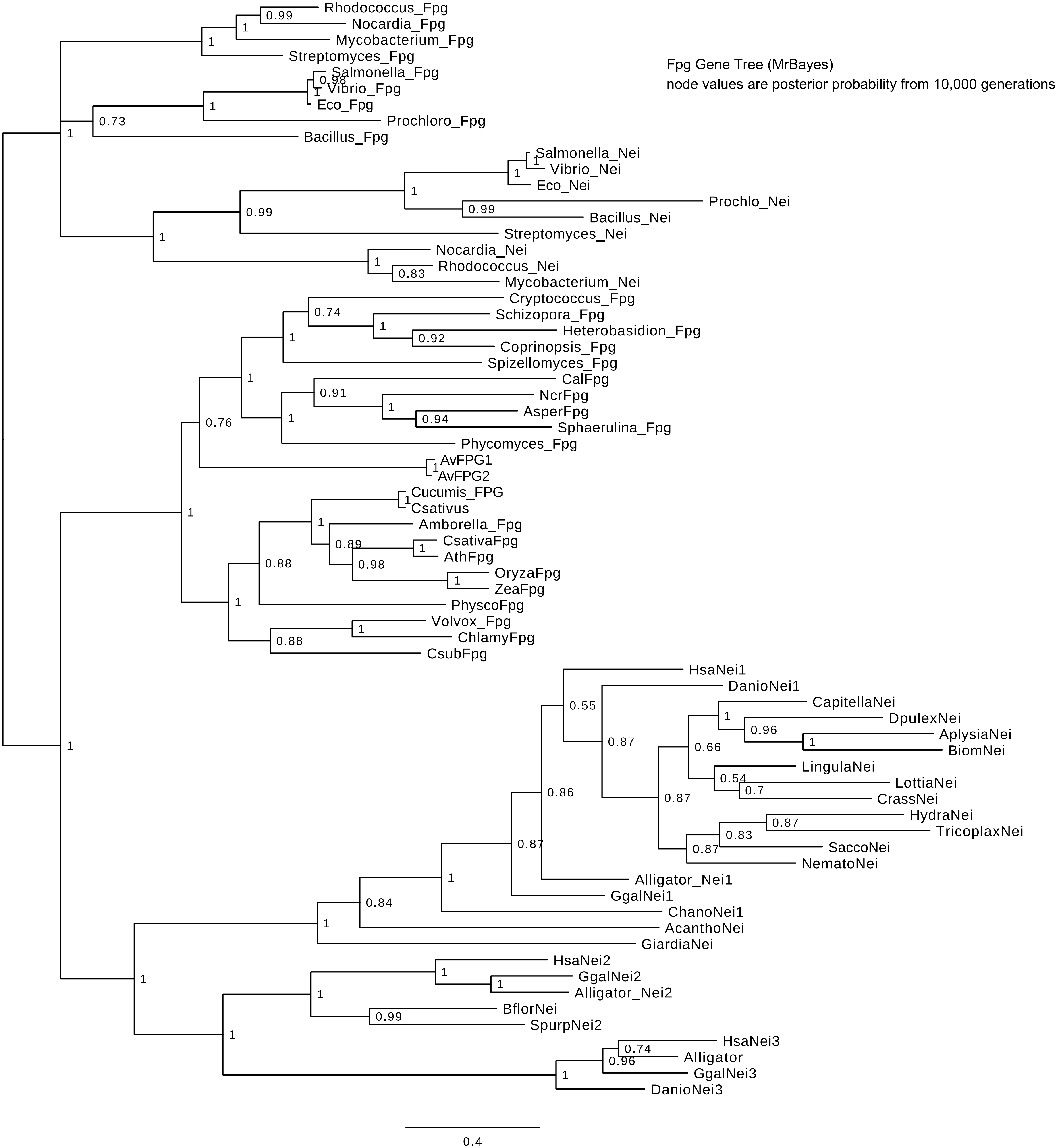
Fpg Gene Tree (MrBayes)
node values are posterior probability from 10,000 generations
Rhodococcus_Fpg
Nocardia_Fpg
Mycobacterium_Fpg
Streptomyces_Fpg
Salmonella_Fpg
Vibrio_Fpg
Eco_Fpg
Prochloro_Fpg
Bacillus_Fpg
Salmonella_Nei
Vibrio_Nei
Eco_Nei
Prochlo_Nei
Bacillus_Nei
Streptomyces_Nei
Nocardia_Nei
Rhodococcus_Nei
Mycobacterium_Nei
Cryptococcus_Fpg
Schizopora_Fpg
Heterobasidion_Fpg
Coprinopsis_Fpg
Spizellomyces_Fpg
CalFpg
NcrFpg
AsperFpg
Sphaerulina_Fpg
Phycomyces_Fpg
AvFPG1
AvFPG2
Cucumis_FPG
Csativus
Amborella_Fpg
CsativaFpg
AthFpg
OryzaFpg
ZeaFpg
PhyscoFpg
Volvox_Fpg
ChlamyFpg
CsubFpg
HsaNei1
DanioNei1
CapitellaNei
DpulexNei
AplysiaNei
BiomNei
LingulaNei
LottiaNei
CrassNei
HydraNei
TricoplaxNei
SaccoNei
NematoNei
Alligator_Nei1
GgalNei1
ChanoNei1
AcanthoNei
GiardiaNei
HsaNei2
GgalNei2
Alligator_Nei2
BflorNei
SpurpNei2
HsaNei3
Alligator
GgalNei3
DanioNei3
0.99
1
1
0.98
1
1
0.73
1
1
1
0.99
1
0.99
1
1
0.83
0.74
1
0.92
1
1
0.91
1
0.94
1
0.76
1
1
1
1
0.89
1
0.98
1
0.88
1
1
0.88
0.55
1
0.96
1
0.66
0.54
0.7
0.87
0.87
0.87
0.83
0.87
0.86
0.87
1
0.84
1
1
1
1
1
0.99
0.74
0.96
1
1
0.4

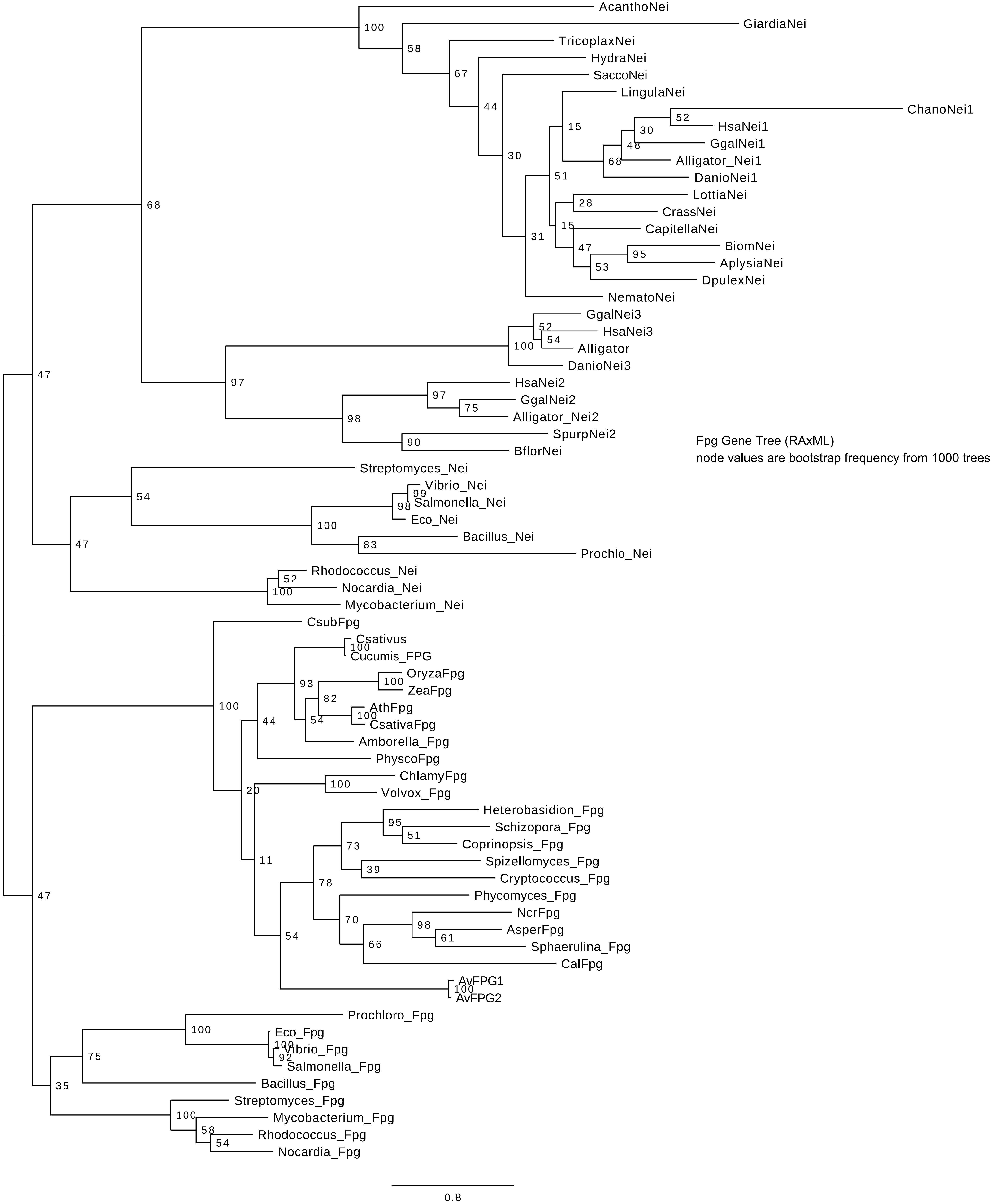
AcanthoNei
GiardiaNei
TricoplaxNei
HydraNei
SaccoNei
LingulaNei
ChanoNei1
HsaNei1
GgalNei1
Alligator_Nei1
DanioNei1
LottiaNei
CrassNei
CapitellaNei
BiomNei
AplysiaNei
DpulexNei
NematoNei
GgalNei3
HsaNei3
Alligator
DanioNei3
HsaNei2
GgalNei2
Alligator_Nei2
SpurpNei2
BflorNei
Fpg Gene Tree (RAxML)
node values are bootstrap frequency from 1000 trees
Streptomyces_Nei
Vibrio_Nei
Salmonella_Nei
Eco_Nei
Bacillus_Nei
Prochlo_Nei
Rhodococcus_Nei
Nocardia_Nei
Mycobacterium_Nei
CsubFpg
Csativus
Cucumis_FPG
OryzaFpg
ZeaFpg
AthFpg
CsativaFpg
Amborella_Fpg
PhyscoFpg
ChlamyFpg
Volvox_Fpg
Heterobasidion_Fpg
Schizopora_Fpg
Coprinopsis_Fpg
Spizellomyces_Fpg
Cryptococcus_Fpg
Phycomyces_Fpg
NcrFpg
AsperFpg
Sphaerulina_Fpg
CalFpg
AvFPG1
AvFPG2
Prochloro_Fpg
Eco_Fpg
Vibrio_Fpg
Salmonella_Fpg
Bacillus_Fpg
Streptomyces_Fpg
Mycobacterium_Fpg
Rhodococcus_Fpg
Nocardia_Fpg
0.8

Fpg		
OTU Name	Accession	Species Name
AcanthoNei	XP_004338875.1	Acanthamoeba castellanii
Alligator Nei3	XP_019336904.1	Alligator mississippiensis
Alligator_Nei1	XP_006265499.1	Alligator mississippiensis
Alligator_Nei2	XP_006274892.2	Alligator mississippiensis
Amborella_Fpg	XP_006856551.1	Amborella trichopoda
AplysiaNei	XP_005108711.1	Aplysia californica
AsperFpg	XP_001820131.1	Aspergillus oryzae
AthFpg	NP_564608.1	Arabidopsis thaliana
AvFPG1	iroGSADVT00000203001	Adineta vaga
AvFPG2	iroGSADVT00048885001	Adineta vaga
Bacillus_Fpg	WP_001114480.1	Bacillus anthracis
Bacillus_Nei	WP_013081902.1	Bacillus anthracis
BflorNei	XP_002594913.1	Branchiostoma floridae
BiomNei	XP_013076604.1	Biomphalaria glabrata
CalFpg	KHC32096.1	Candida albicans
CapitellaNei	ELU15622.1	Capitella teleta
ChanoNei1	XP_004994846.1	Salpingoeca rosetta
ChlamyFpg	XP_001702534.1	Chlamydomonas reinhardtii
Coprinopsis_Fpg	XP_001828971.2	Coprinopsis cinerea okayama
CrassNei	XP_011418153.1	Crassostrea gigas
Cryptococcus_Fpg	XP_572248.1	Cryptococcus neoformans
CsativaFpg	XP_010500852.1	Camelina sativa
Csativus	XP_011652995.1	Cucumis sativus
CsubFpg	XP_005649162.1	Coccomyxa subellipsoidea
Cucumis_FPG	XP_008454183.1	Cucumis melo
DanioNei1	NP_956577.2	Danio rerio
DanioNei3	AAI55158.1	Danio rerio
DpulexNei	EFX74223.1	Daphnia pulex
Eco_Fpg	EJE96814.1	Escherichia coli
Eco_Nei	WP_001113989.1	Escherichia coli
GgalNei1	NP_001074345.1	Gallus gallus
GgalNei2	XP_015140617.1	Gallus gallus
GgalNei3	XP_426306.3	Gallus gallus
GiardiaNei	EET02426.1	Giardia intestinalis
Heterobasidion_Fpg	XP_009541575.1	Heterobasidion irregulare
HsaNei1	NP_001243481.1	Homo sapiens
HsaNei2	NP_659480.1	Homo sapiens
HsaNei3	NP_060718.2	Homo sapiens
HydraNei	XP_012557757.1	Hydra vulgaris
LingulaNei	XP_013389918.1	Lingula anatina
LottiaNei	XP_009061718.1	Lottia gigantea
Mycobacterium_Fpg	WP_003414814.1	Mycobacterium tuberculosis
Mycobacterium_Nei	WP_003412657.1	Mycobacterium tuberculosis
NcrFpg	XP_958637.2	Neurospora crassa
NematoNei	XP_001629836.1	Nematostella vectensis
Nocardia_Fpg	WP_014352116.1	Nocardia cyriacigeorgica
Nocardia_Nei	WP_036534381.1	Nocardia cyriacigeorgica
OryzaFpg	XP_015641389.1	Oryza sativa
Phycomyces_Fpg	XP_018291457.1	Phycomyces blakesleeanus
PhyscoFpg	XP_001782579.1	Physcomitrella patens
Prochlo_Nei	WP_011823937.1	Prochlorococcus sp.
Prochloro_Fpg	WP_025936373.1	Prochlorococcus sp.
Rhodococcus_Fpg	WP_039586931.1	Rhodococcus opacus
Rhodococcus_Nei	WP_012688319.1	Rhodococcus opacus
SaccoNei	XP_006818873.1	Saccoglossus kowalevskii
Salmonella_Fpg	WP_001114507.1	Salmonella enterica
Salmonella_Nei	WP_053445184.1	Salmonella enterica
Schizopora_Fpg	KLO05707.1	Schizopora paradoxa
Sphaerulina_Fpg	XP_016765645.1	Sphaerulina musiva
Spizellomyces_Fpg	KNC96931.1	Spizellomyces punctatus
SpurpNei2	XP_011660809.1	Strongylocentrotus purpuratus
Streptomyces_Fpg	WP_003961998.1	Streptomyces clavuligerus
Streptomyces_Nei	WP_003962081.1	Streptomyces clavuligerus
TricoplaxNei	XP_002110397.1	Trichoplax adhaerens
Vibrio_Fpg	WP_044328861.1	Vibrio parahaemolyticus
Vibrio_Nei	WP_042781308.1	Vibrio parahaemolyticus
Volvox_Fpg	XP_002954128.1	Volvox carteri
ZeaFpg	NP_001141023.1	Zea mays

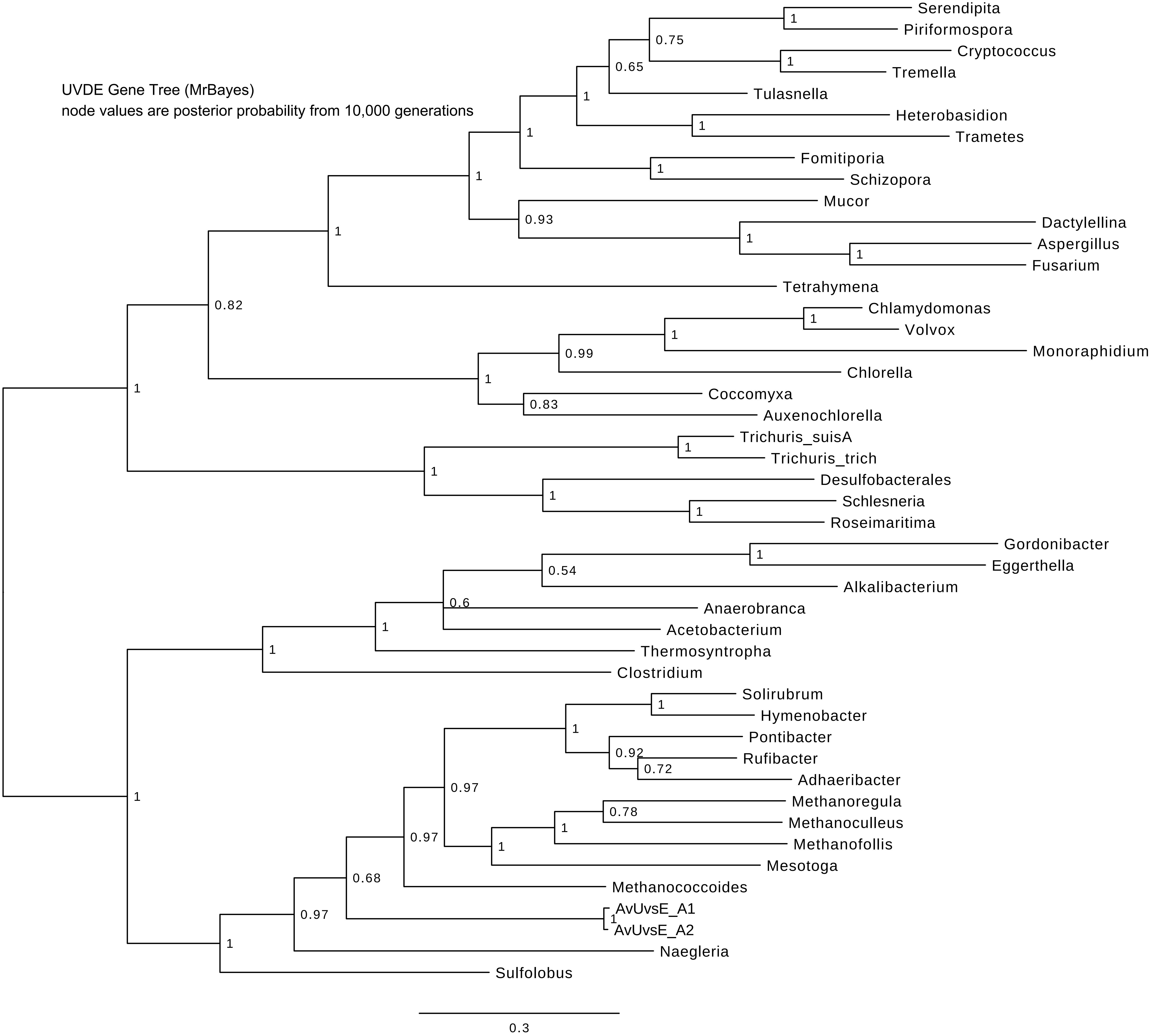

UVDE Gene Tree (MrBayes)
node values are posterior probability from 10,000 generations
Serendipita
Piriformospora
Cryptococcus
Tremella
Tulasnella
Heterobasidion
Trametes
Fomitiporia
Schizopora
Mucor
Dactylellina
Aspergillus
Fusarium
Tetrahymena
Chlamydomonas
Volvox
Monoraphidium
Chlorella
Coccomyxa
Auxenochlorella
Trichuris_suisA
Trichuris_trich
Desulfobacterales
Schlesneria
Roseimaritima
Gordonibacter
Eggerthella
Alkalibacterium
Anaerobranca
Acetobacterium
Thermosyntropha
Clostridium
Solirubrum
Hymenobacter
Pontibacter
Rufibacter
Adhaeribacter
Methanoregula
Methanoculleus
Methanofollis
Mesotoga
Methanococcoides
AvUvsE_A1
AvUvsE_A2
Naegleria
Sulfolobus
0.75
0.65
0.93
0.82
0.99
0.83
0.54
0.6
0.92
0.72
0.97
0.78
0.97
0.68
0.97
0.3

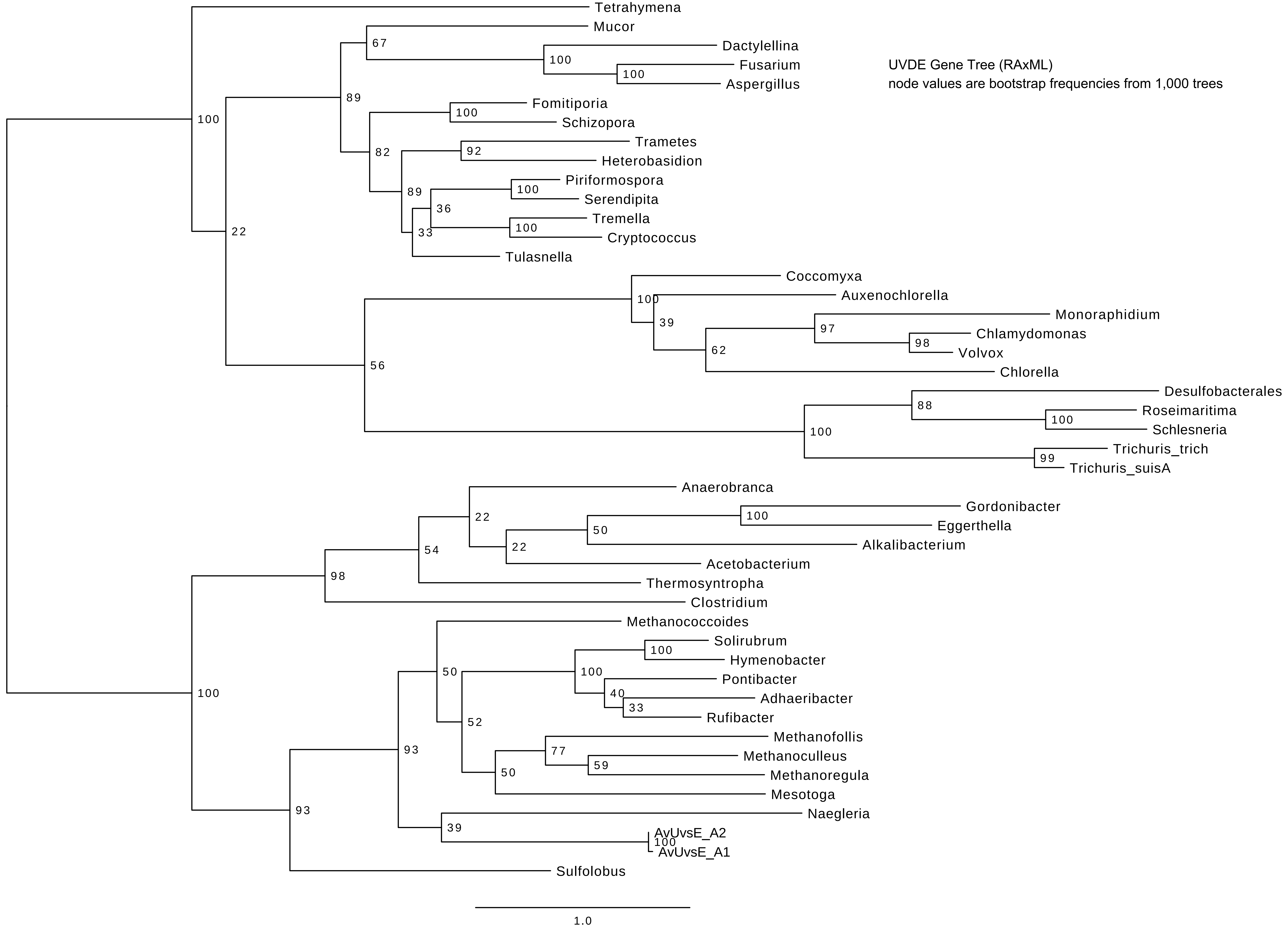
UVDE Gene Tree (RAxML)
node values are bootstrap frequencies from 1,000 trees
Tetrahymena
Mucor
Dactylellina
Fusarium
Aspergillus
Fomitiporia
Schizopora
Trametes
Heterobasidion
Piriformospora
Serendipita
Tremella
Cryptococcus
Tulasnella
Coccomyxa
Auxenochlorella
Monoraphidium
Chlamydomonas
Volvox
Chlorella
Desulfobacterales
Roseimaritima
Schlesneria
Trichuris_trich
Trichuris_suisA
Anaerobranca
Gordonibacter
Eggerthella
Alkalibacterium
Acetobacterium
Thermosyntropha
Clostridium
Methanococcoides
Solirubrum
Hymenobacter
Pontibacter
Adhaeribacter
Rufibacter
Methanofollis
Methanoculleus
Methanoregula
Mesotoga
Naegleria
AvUvsE_A2
AvUvsE_A1
Sulfolobus
1.0

UVDE

OTU Name	Accession	Species Name
Acetobacterium	WP_026395692.1	Acetobacterium dehalogenans
Adhaeribacter	WP_026463679.1	Adhaeribacter aquaticus
Alkalibacterium	WP_034302136.1	Alkalibacterium sp. AK22
Anaerobranca	WP_072908311.1	Anaerobranca californiensis
Aspergillus	GAA88011.1	Aspergillus kawachii IFO 4308
Auxenochlorella	XP_011397296.1	Auxenochlorella protothecoides
AvUvsE_A1	GSADVT00018071001	Adineta vaga
AvUvsE_A2	GSADVT00014593001	Adineta vaga
Chlamydomonas	XP_001690812.1	Chlamydomonas reinhardtii
Chlorella	XP_005846255.1	Chlorella variabilis
Clostridium	WP_003463088.1	Clostridium perfringens
Coccomyxa	XP_005646853.1	Coccomyxa subellipsoidea C-169
Cryptococcus	AGW24517.1	Cryptococcus neoformans var. neoformans
Dactylellina	XP_011112666.1	Dactylellina haptotyla CBS 200.50
Desulfobacterales	KPK26905.1	Desulfobacterales bacterium SG8_35_2
Eggerthella	WP_009608514.1	Eggerthella
Fomitiporia	XP_007271059.1	Fomitiporia mediterranea MF3/22
Fusarium	EXK76798.1	Fusarium oxysporum f. sp. raphani 54005
Gordonibacter	WP_041238780.1	Gordonibacter pamelaeae
Heterobasidion	XP_009550464.1	Heterobasidion irregulare TC 32-1
Hymenobacter	WP_044512026.1	Hymenobacter sp. DG25B
Mesotoga	WP_014731440.1	Mesotoga prima
Methanococcoides	WP_048204699.1	Methanococcoides methylutens MM1
Methanoculleus	WP_014866629.1	Methanoculleus bourgensis
Methanofollis	WP_004039194.1	Methanofollis liminatans
Methanoregula	WP_015286025.1	Methanoregula formicica
Monoraphidium	XP_013896386.1	Monoraphidium neglectum
Mucor	EPB87176.1	Mucor circinelloides f. circinelloides 1006PhL
Naegleria	XP_002674735.1	Naegleria gruberi
Piriformospora	CCA75708.1	Piriformospora indica DSM 11827
Pontibacter	WP_025607992.1	Pontibacter actiniarum
Roseimaritima	WP_068140912.1	Roseimaritima ulvae
Rufibacter	WP_048921566.1	Rufibacter sp. DG31D
Schizopora	KLO08210.1	Schizopora paradoxa
Schlesneria	WP_010585944.1	Schlesneria paludicola
Serendipita	KIM27248.1	Serendipita vermifera MAFF 305830
Solirubrum	WP_059067335.1	Solirubrum puertoriconensis
Sulfolobus	WP_011277952.1	Sulfolobus acidocaldarius DSM 639
Tetrahymena	XP_001022149.2	Tetrahymena thermophila SB210
Thermosyntropha	WP_073090394.1	Thermosyntropha lipolytica
Trametes	CDO70055.1	Trametes cinnabarina
Tremella	XP_007002301.1	Tremella mesenterica DSM 1558
Trichuris_suisA	KHJ48376.1	Trichuris suis
Trichuris_trich	CDW54875.1	Trichuris trichiura
Tulasnella	KIO21278.1	Tulasnella calospora MUT 4182
Volvox	XP_002948125.1	Volvox carteri f. nagariensis

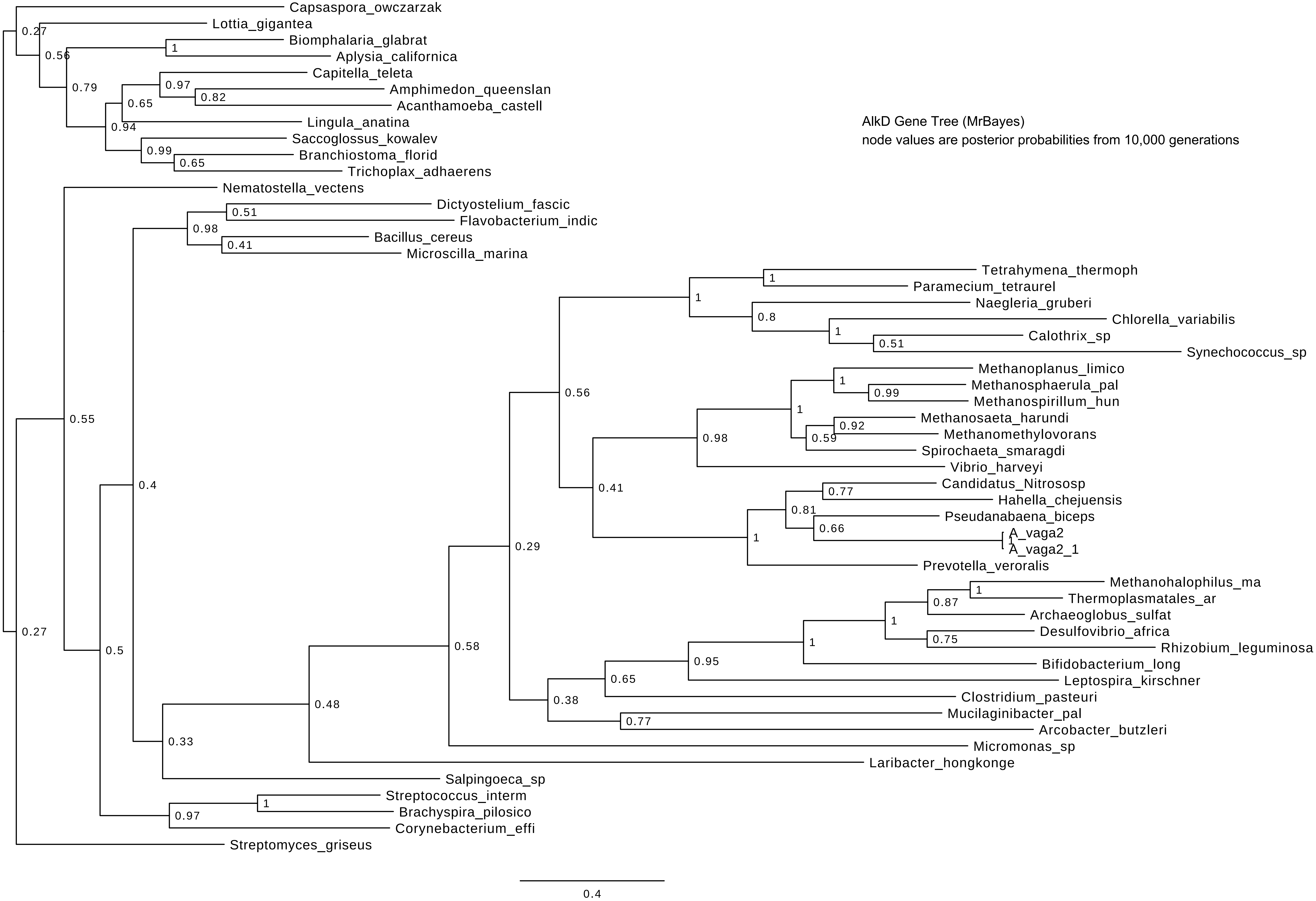
AlkD Gene Tree (MrBayes)
node values are posterior probabilities from 10,000 generations
Capsaspora_owczarzak
Lottia_gigantea
Biomphalaria_glabrat
Aplysia_californica
Capitella_teleta
Amphimedon_queenslan
Acanthamoeba_castell
Lingula_anatina
Saccoglossus_kowalev
Branchiostoma_florid
Trichoplax_adhaerens
Nematostella_vectens
Dictyostelium_fascic
Flavobacterium_indic
Bacillus_cereus
Microscilla_marina
Tetrahymena_thermoph
Paramecium_tetraurel
Naegleria_gruberi
Chlorella_variabilis
Calothrix_sp
Synechococcus_sp
Methanoplanus_limico
Methanosphaerula_pal
Methanospirillum_hun
Methanosaeta_harundi
Methanomethylovorans
Spirochaeta_smaragdi
Vibrio_harveyi
Candidatus_Nitrososp
Hahella_chejuensis
Pseudanabaena_biceps
A_vaga2
A_vaga2_1
Prevotella_veroralis
Methanohalophilus_ma
Thermoplasmatales_ar
Archaeoglobus_sulfat
Desulfovibrio_africa
Rhizobium_leguminosa
Bifidobacterium_long
Leptospira_kirschner
Clostridium_pasteuri
Mucilaginibacter_pal
Arcobacter_butzleri
Micromonas_sp
Laribacter_hongkonge
Salpingoeca_sp
Streptococcus_interm
Brachyspira_pilosico
Corynebacterium_effi
Streptomyces_griseus
0.4

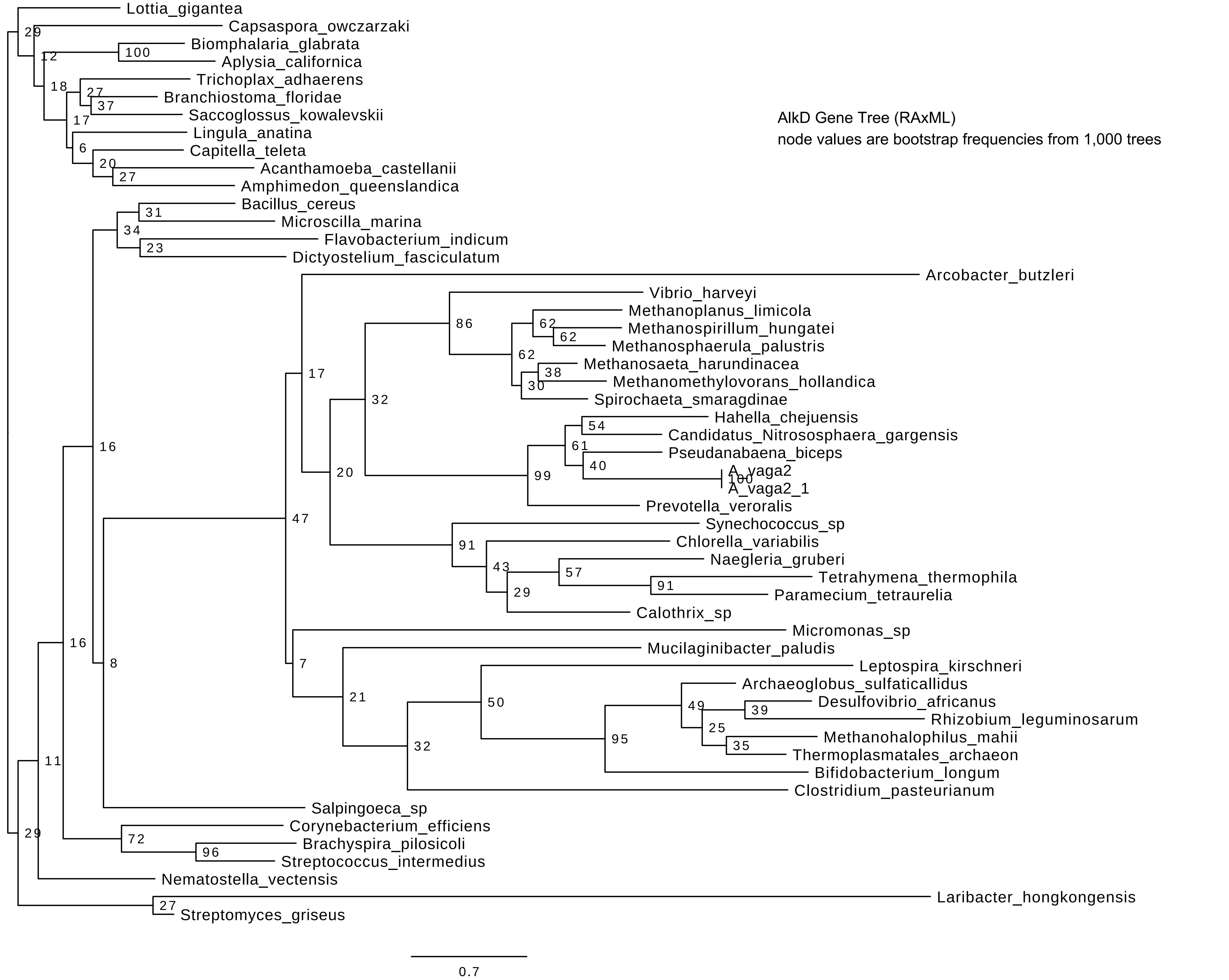

AlkD Gene Tree (RAxML)
node values are bootstrap frequencies from 1,000 trees
Lottia_gigantea
Capsaspora_owczarzaki
Biomphalaria_glabrata
Aplysia_californica
Trichoplax_adhaerens
Branchiostoma_floridae
Saccoglossus_kowalevskii
Lingula_anatina
Capitella_teleta
Acanthamoeba_castellanii
Amphimedon_queenslandica
Bacillus_cereus
Microscilla_marina
Flavobacterium_indicum
Dictyostelium_fasciculatum
Arcobacter_butzleri
Vibrio_harveyi
Methanoplanus_limicola
Methanospirillum_hungatei
Methanosphaerula_palustris
Methanosaeta_harundinacea
Methanomethylovorans_hollandica
Spirochaeta_smaragdinae
Hahella_chejuensis
Candidatus_Nitrososphaera_gargensis
Pseudanabaena_biceps
A_vaga2
A_vaga2_1
Prevotella_veroralis
Synechococcus_sp
Chlorella_variabilis
Naegleria_gruberi
Tetrahymena_thermophila
Paramecium_tetraurelia
Calothrix_sp
Micromonas_sp
Mucilaginibacter_paludis
Leptospira_kirschneri
Archaeoglobus_sulfaticallidus
Desulfovibrio_africanus
Rhizobium_leguminosarum
Methanohalophilus_mahii
Thermoplasmatales_archaeon
Bifidobacterium_longum
Clostridium_pasteurianum
Salpingoeca_sp
Corynebacterium_efficiens
Brachyspira_pilosicoli
Streptococcus_intermedius
Nematostella_vectensis
Laribacter_hongkongensis
Streptomyces_griseus
0.7

AlkD

OTU Name	Accession	Species Name
A_vagaA1	GSADVT00061821001	Adineta vaga
A_vagaA2	GSADVT00053068001	Adineta vaga
Acanthamoeba_castellanii	XP_004334680.1	Acanthamoeba castellanii
Amphimedon_queenslandica	XP_011405924.1	Amphimedon queenslandica
Aplysia_californica	XP_005095911.1	Aplysia californica
Archaeoglobus_sulfaticallidus	WP_015591307.1	Archaeoglobus sulfaticallidus
Arcobacter_butzleri	EFU68900.1	Arcobacter butzleri
Bacillus_cereus	CAJ31885.1	Bacillus cereus
Bifidobacterium_longum	EIJ28085.1	Bifidobacterium longum
Biomphalaria_glabrata	XP_013069563.1	Biomphalaria glabrata
Brachyspira_pilosicoli	WP_013245028.1	Brachyspira pilosicoli
Branchiostoma_floridae	XP_002585934.1	Branchiostoma floridae
Calothrix_sp	WP_015198066.1	Calothrix sp
Candidatus_Nitrososphaera_gargensis	AFU59935.1	Candidatus Nitrososphaera
Capitella_teleta	ELU03766.1	Capitella teleta
Capsaspora_owczarzaki	KJE92821.1	Capsaspora owczarzaki
Chlorella_variabilis	XP_005846594.1	Chlorella variabilis
Clostridium_pasteurianum	ELP61122.1	Clostridium pasteurianum
Corynebacterium_efficiens	WP_006770023.1	Corynebacterium efficiens
Desulfovibrio_africanus	WP_005988493.1	Desulfovibrio africanus
Dictyostelium_fasciculatum	XP_004355281.1	Dictyostelium fasciculatum
Flavobacterium_indicum	WP_014388571.1	Flavobacterium indicum
Hahella_chejuensis	WP_011399168.1	Hahella chejuensis
Laribacter_hongkongensis	WP_052292614.1	Laribacter hongkongensis
Leptospira_kirschneri	WP_004777096.1	Leptospira kirschneri
Lingula_anatina	XP_013421605.1	Lingula anatina
Lottia_gigantea	XP_009064864.1	Lottia gigantea
Methanohalophilus_mahii	WP_013036927.1	Methanohalophilus mahii
Methanomethylovorans_hollandica	WP_015323966.1	Methanomethylovorans hollandica
Methanoplanus_limicola	EHQ36038.1	Methanoplanus limicola
Methanosaeta_harundinacea	AET64962.1	Methanosaeta harundinacea
Methanosphaerula_palustris	WP_012618312.1	Methanosphaerula palustris
Methanospirillum_hungatei	ABD41293.1	Methanospirillum hungatei
Micromonas_sp	XP_002508650.1	Micromonas sp
Microscilla_marina	WP_004156026.1	Microscilla marina
Mucilaginibacter_paludis	WP_008511893.1	Mucilaginibacter paludis
Naegleria_gruberi	XP_002668790.1	Naegleria gruberi
Nematostella_vectensis	XP_001641053.1	Nematostella vectensis
Paramecium_tetraurelia	XP_001424901.1	Paramecium tetraurelia
Prevotella_veroralis	WP_004383329.1	Prevotella veroralis
Pseudanabaena_biceps	WP_009627758.1	Pseudanabaena biceps
Rhizobium_leguminosarum	WP_003594087.1	Rhizobium leguminosarum
Saccoglossus_kowalevskii	XP_002741717.1	Saccoglossus kowalevskii
Salpingoeca_sp	XP_004990973.1	Salpingoeca sp
Spirochaeta_smaragdinae	WP_013252727.1	Spirochaeta smaragdinae
Streptococcus_intermedius	BAM23676.1	Streptococcus intermedius
Streptomyces_griseus	BAG23066.1	Streptomyces griseus
Synechococcus_sp	WP_006454027.1	Synechococcus sp
Tetrahymena_thermophila	XP_001017755.2	Tetrahymena thermophila
Thermoplasmatales_archaeon	ENO11913.1	Thermoplasmatales archaeon
Trichoplax_adhaerens	XP_002108671.1	Trichoplax adhaerens
Vibrio_harveyi	WP_005427786.1	Vibrio harveyi

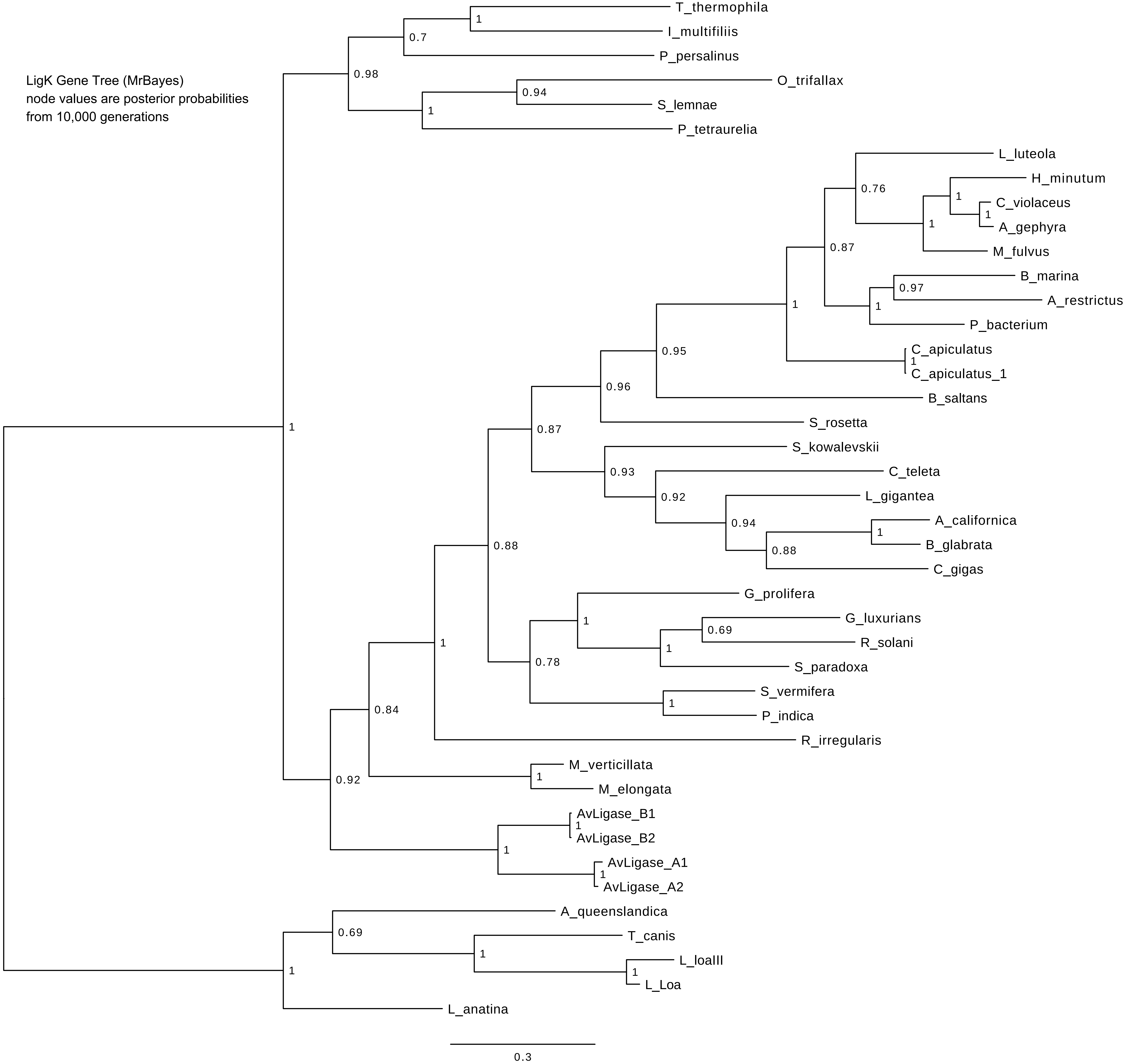
LigK Gene Tree (MrBayes)
node values are posterior probabilities
from 10,000 generations
T_thermophila
I_multifiliiis
P_persalinus
O_trifallax
S_lemnae
P_tetraurelia
L_luteola
H_minutum
C_violaceus
A_gephyra
M_fulvus
B_marina
A_restrictus
P_bacterium
C_apiculatus
C_apiculatus_1
B_saltans
S_rosetta
S_kowalevskii
C_teleta
L_gigantea
A_californica
B_glabrata
C_gigas
G_prolifera
G_luxurians
R_solani
S_paradoxa
S_vermifera
P_indica
R_irregularis
M_verticillata
M_elongata
AvLigase_B1
AvLigase_B2
AvLigase_A1
AvLigase_A2
A_queenslandica
T_canis
L_loaIII
L_Loa
L_anatina
0.3

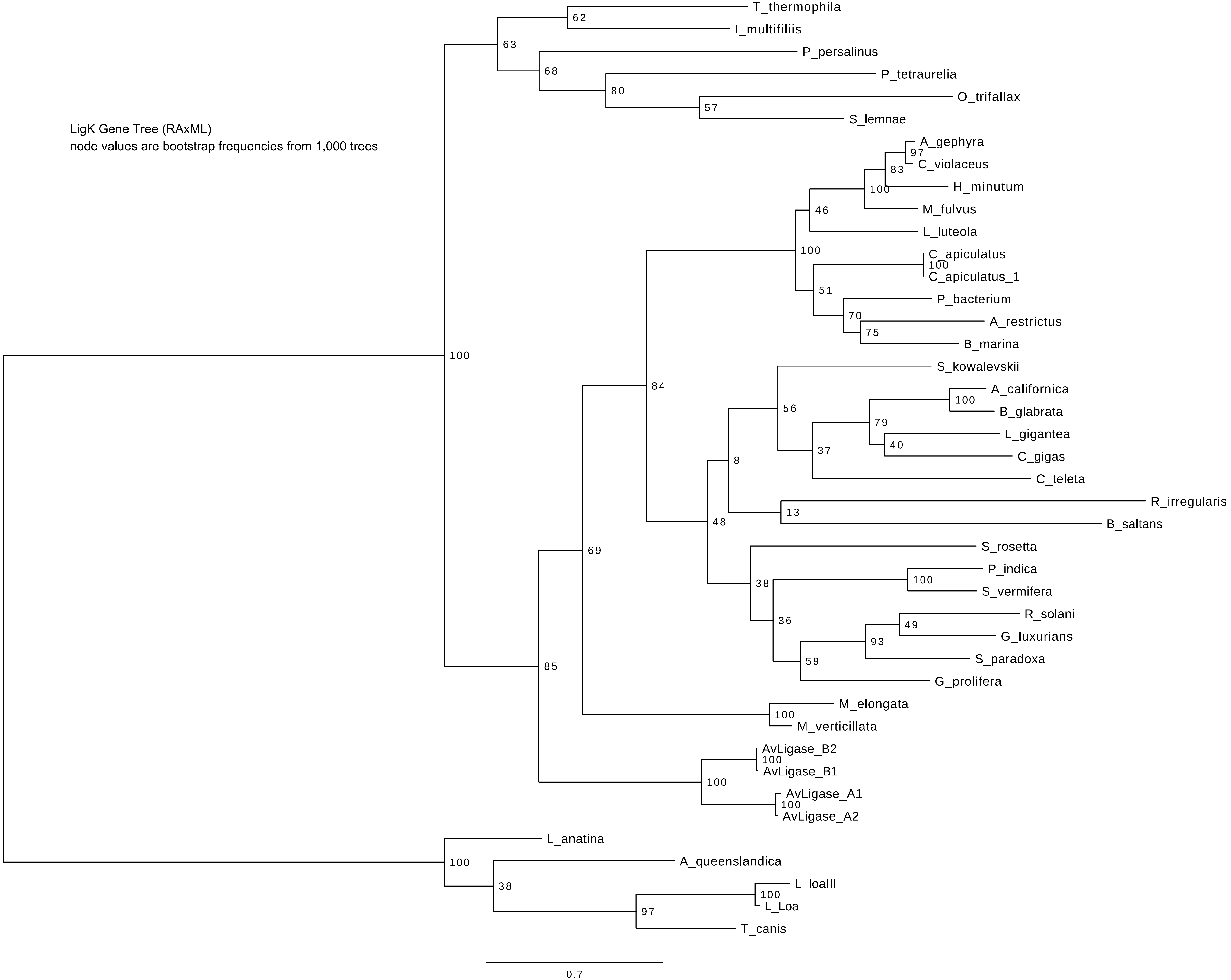
LigK Gene Tree (RAxML)
node values are bootstrap frequencies from 1,000 trees
T_thermophila
I_multifiliis
P_persalinus
P_tetraurelia
O_trifallax
S_lemnae
A_gephyra
C_violaceus
H_minutum
M_fulvus
L_luteola
C_apiculatus
C_apiculatus_1
P_bacterium
A_restrictus
B_marina
S_kowalevskii
A_californica
B_glabrata
L_gigantea
C_gigas
C_teleta
R_irregularis
B_saltans
S_rosetta
P_indica
S_vermifera
R_solani
G_luxurians
S_paradoxa
G_prolifera
M_elongata
M_verticillata
AvLigase_B2
AvLigase_B1
AvLigase_A1
AvLigase_A2
L_anatina
A_queenslandica
L_loaIII
L_Loa
T_canis
0.7

LigK

OTU Name	Accession	Species Name
A_californica	XP_005100834.1	Aplysia californica
A_gephyra	WP_047858784.1	Archangium gephyra
A_queenslandica	XP_011410314.1	Amphimedon queenslandica
A_restrictus	WP_026685685.1	Azovibrio restrictus
AvLigase_A1	GSADVT00019625001	Adineta vaga
AvLigase_A2	GSADVT00027869001	Adineta vaga
AvLigase_B1	GSADVT00042987002	Adineta vaga
AvLigase_B2	GSADVT00047807001	Adineta vaga
B_glabrata	XP_013093455.1	Biomphalaria glabrata
B_marina	WP_002650560.1	Blastopirellula marina
B_saltans	CUE93864.1	Bodo saltans
C_apiculatus	WP_044240733.1	Chondromyces apiculatus
C_apiculatus_1	WP_044240733.1	Chondromyces apiculatus
C_gigas	XP_011433142.1	Crassostrea gigas
C_teleta	ELT89513.1	Capitella teleta
C_violaceus	WP_043389891.1	Cystobacter violaceus
G_luxurians	KIK57446.1	Gymnopus luxurians
G_prolifera	KXS17784.1	Gonapodya prolifera
H_minutum	WP_044188268.1	Hyalangium minutum
I_multifiliis	XP_004037347.1	Ichthyophthirius multifiliis
L_anatina	XP_013393552.1	Lingula anatina
L_gigantea	XP_009061413.1	Lottia gigantea
L_Loa	EJD74156.1	Loa loa
L_loaII	XP_003147982.1	Loa loa
L_luteola	AKU94568.1	Labilithrix luteola
M_elongata	OAQ23872.1	Mortierella elongata
M_fulvus	WP_013936241.1	Myxococcus fulvus
M_verticillata	KFH62561.1	Mortierella verticillata
O_trifallax	EJY73190.1	Oxytricha trifallax
P_bacterium	OAI47765.1	Planctomycetaceae bacterium
P_indica	CCA71483.1	Piriformospora indica
P_persalinus	KRX09813.1	Pseudocohnilembus persalinus
P_tetraurelia	XP_001460273.1	Paramecium tetraurelia strain
R_irregularis	ESA15105.1	Rhizophagus irregularis
R_solani	EUC59263.1	Rhizoctonia solani
S_kowalevskii	XP_002737951.1	Saccoglossus kowalevskii
S_lemnae	CDW80774.1	Stylonychia lemnae
S_paradoxa	KLO11458.1	Schizopora paradoxa
S_rosetta	XP_004993722.1	Salpingoeca rosetta
S_vermifera	KIM28348.1	Serendipita vermifera
T_canis	KHN83735.1	Toxocara canis
T_thermophila	XP_001011861.1	Tetrahymena thermophila
