# Supplementary material for: Evolutionary diversity and novelty of DNA repair genes in asexual Bdelloid rotifers
Source: BMC Evol Biol. 2018 Nov 28;18:177. doi: 10.1186/s12862-018-1288-9 (PMC6264785; doi:10.1186/s12862-018-1288-9)
Supplement: Supplementary file 3 — Script for RNASeq Analysis. This Word file contains a bash script to run the rsem mapping and differential gene expression tests. (DOCX 113 kb) [file 12862_2018_1288_MOESM3_ESM.docx]

**Supplemental Methods**

This is a bash script to run the rsem mapping and differential gene expression tests

#!/bin/bash

# Bowtie2 v2.3.2

# RSEM 1.3.0

# EBSeq 1.2.0

# clusterize is an in-house script that writes an SGE cluster submission script from the information on the command line

GTF="./Avaga_bhl.gtf"

REF="/workspace/markwelchlab/HER2015/DDR_ImprovedCDS.fa"

# Prepare the reference

rsem-prepare-reference --bowtie2 $REF AvagaHER

# Calculate expression

for i in Hyd Ent Rec

do

for j in 1 2 3

do

clusterize \

rsem-calculate-expression --bowtie2 --num-threads 4 --forward-prob 0 \

--paired-end ../${i}/${i}/minoche/minoche${i}${j}-QUALITY_PASSED_R1.fastq \

../${i}/${i}/minoche/minoche${i}${j}-QUALITY_PASSED_R2.fastq \

AvagaHER ${i}${j}

done

done

# Exit here until rsem finished

#exit

# generate ng vector files

rsem-generate-ngvector AvagaHER.transcripts.fa AvagaHER

# create matrices

rsem-generate-data-matrix Hyd1.genes.results Hyd2.genes.results Hyd3.genes.results \

Ent1.genes.results Ent2.genes.results Ent3.genes.results \

> Hyd_v_Ent.matrix

rsem-generate-data-matrix Ent1.genes.results Ent2.genes.results Ent3.genes.results \

Rec1.genes.results Rec2.genes.results Rec3.genes.results \

> Ent_v_Rec.matrix

rsem-generate-data-matrix Hyd1.genes.results Hyd2.genes.results Hyd3.genes.results \

Rec1.genes.results Rec2.genes.results Rec3.genes.results \

> Hyd_v_Rec.matrix

rsem-generate-data-matrix Hyd1.genes.results Hyd2.genes.results Hyd3.genes.results \

Ent1.genes.results Ent2.genes.results Ent3.genes.results \

Rec1.genes.results Rec2.genes.results Rec3.genes.results \

> Hyd_v_Ent_v_Rec.matrix

# run EBSeq

rsem-run-ebseq Hyd_v_Ent.matrix 3,3 Hyd_v_Ent.ebseq &

rsem-run-ebseq Ent_v_Rec.matrix 3,3 Ent_v_Rec.ebseq &

rsem-run-ebseq Hyd_v_Rec.matrix 3,3 Hyd_v_Rec.ebseq &

rsem-run-ebseq Hyd_v_Ent_v_Rec.matrix 3,3,3 Hyd_v_Ent_v_Rec.ebseq &

# run edgeR|DESeq2|voom|ROTS

# conditions files need to be generated by hand

PROG="/bioware/trinityrnaseq/Analysis/DifferentialExpression/run_DE_analysis.pl"

for i in Hyd_v_Ent Hyd_v_Rec Ent_v_Rec

do

for j in DESeq2 edgeR voom

do

$PROG --matrix $i.matrix --output ${i}_${j} \

--method $j --samples_file conditions_$i &

done

done

This is an example of R history to check the size factor of the geometric mean

library(DESeq)

Matrix = read.table("Hyd_v_Ent.matrix.txt", header=TRUE, row.names=1)

condition = factor(c("hyd","hyd","hyd","rec","rec","rec"))

cds = newCountDataSet(Matrix,condition)

geomeans <- exp(rowMeans(log(counts(cds))))

print( sizeFactors(cds)[j] )

j <- 1

hist(log2(counts(cds)[,j]/geomeans),breaks=100)

abline( v=log2( sizeFactors(cds)[ j ] ), col="red" )

sizeFactors(cds)

j <- 2

hist(log2(counts(cds)[,j]/geomeans),breaks=100)

abline( v=log2( sizeFactors(cds)[ j ] ), col="red" )

sizeFactors(cds)
